# Administration of Linoleoylethanolamide Reduced Weight Gain, Dyslipidemia, and Inflammation Associated with High-Fat-Diet-Induced Obesity

**DOI:** 10.3390/nu15204448

**Published:** 2023-10-20

**Authors:** Rubén Tovar, Marialuisa de Ceglia, Massimo Ubaldi, Miguel Rodríguez-Pozo, Laura Soverchia, Carlo Cifani, Gema Rojo, Ana Gavito, Laura Hernandez-Folgado, Nadine Jagerovic, Roberto Ciccocioppo, Elena Baixeras, Fernando Rodríguez de Fonseca, Juan Decara

**Affiliations:** 1 Grupo de Neuropsicofarmacología, Instituto IBIMA-Plataforma BIONAND, Unidad de Gestión Clínica de Salud Mental, Hospital Regional Universitario de Málaga, Avda, Carlos Haya 82, Sótano, 29010 Málaga, Spain; rubentovar7@hotmail.com (R.T.); marialuisa.deceglia@ibima.eu (M.d.C.); rpmiguel@uma.es (M.R.-P.); analugavito@hotmail.com (A.G.); ebaixeras@uma.es (E.B.); 2 School of Pharmacy, Pharmacology Unit, University of Camerino, 62032 Camerino, Italy; massimo.ubaldi@unicam.it (M.U.); laura.soverchia@unicam.it (L.S.); carlo.cifani@unicam.it (C.C.); roberto.ciccocioppo@unicam.it (R.C.); 3 Department of Endocrinology and Nutrition, Hospital Regional Universitario de Málaga, Instituto IBIMA-Plataforma BIONAND, 29010 Málaga, Spain; gemma.rojo.m@gmail.com; 4 Instituto de Química Médica, Consejo Superior de Investigaciones Científicas, Avenida Juan de la Cierva, 28006 Madrid, Spain; lauraherf@hotmail.com (L.H.-F.); nadine@iqm.csic.es (N.J.); 5 Unidad Clínica de Neurología, Hospital Regional Universitario de Málaga, Instituto IBMA-Plataforma BIONAND, 29010 Málaga, Spain; 6 Andalusian Network for Clinical and Translational Research in Neurology [NEURO-RECA], 29010 Malaga, Spain

**Keywords:** obesity, liver steatosis, high-fat diet, acylethanolamides, linoleic acid, linoleylethanolamide

## Abstract

Acylethanolamides (NAEs) are bioactive lipids derived from diet fatty acids that modulate important homeostatic functions, including appetite, fatty acid synthesis, mitochondrial respiration, inflammation, and nociception. Among the naturally circulating NAEs, the pharmacology of those derived from either arachidonic acid (Anandamide), oleic acid (OEA), and palmitic acid (PEA) have been extensively characterized in diet-induced obesity. For the present work, we extended those studies to linoleoylethanolamide (LEA), one of the most abundant NAEs found not only in plasma and body tissues but also in foods such as cereals. In our initial study, circulating concentrations of LEA were found to be elevated in overweight humans (body mass index (BMI, Kg/m^2^) > 25) recruited from a representative population from the south of Spain, together with AEA and the endocannabinoid 2-Arachidonoyl glycerol (2-AG). In this population, LEA concentrations correlated with the circulating levels of cholesterol and triglycerides. In order to gain insight into the pharmacology of LEA, we administered it for 14 days (10 mg/kg i.p. daily) to obese male Sprague Dawley rats receiving a cafeteria diet or a standard chow diet for 12 consecutive weeks. LEA treatment resulted in weight loss and a reduction in circulating triglycerides, cholesterol, and inflammatory markers such as Il-6 and Tnf-alpha. In addition, LEA reduced plasma transaminases and enhanced acetyl-CoA-oxidase (Acox) and Uncoupling protein-2 (Ucp2) expression in the liver of the HFD-fed animals. Although the liver steatosis induced by the HFD was not reversed by LEA, the overall data suggest that LEA contributes to the homeostatic signals set in place in response to diet-induced obesity, potentially contributing with OEA to improve lipid metabolism after high fat intake. The anti-inflammatory response associated with its administration suggests its potential for use as a nutrient supplement in non-alcoholic steatohepatitis.

## 1. Introduction

Acylethanolamides (NAEs) are signaling lipids that modulate multiple functions related to body homeostasis, including those related to energy balance, such as appetite, lipid metabolism, and glucose utilization [1,2]. One of the main pathways for NAEs biosynthesis is through the on-demand cleavage of membrane phospholipid precursors, N-acyl-phosphatidylethanolamines (NAPEs). In turn, NAPE biosynthesis is partially regulated by the dietary intake of fatty acids [3,4]. NAEs can be also found in relevant amounts in certain foods, such as cereals and legumes [5], being rapidly released by mastication into the saliva [6]. Additionally, NAEs can be produced actively by gut microbiota [7].

Among the different NAEs detected in plasma, three of them, namely Anandamide (AEA, a cannabinoid Cb1 receptor partial agonist), Oleoylethanolamide (OEA, an agonist of peroxisome proliferator-activated receptor, Ppar-α), and Palmitoylethanolamide (PEA, a multitarget compound capable of interacting with both cannabinoid Cb1 and Cb2 receptors and Ppar-α receptors), are the best characterized [1,2,8,9]. All NAEs are degraded by enzymatic hydrolysis catalyzed by the enzymes fatty acid amidohydrolase (Faah) and N-acylethanolamine acid amidase [9,10]. AEA has been found to be analgetic, to enhance appetite, to act at both central feeding modulatory centers and peripheral nerve terminals, and to shift metabolism towards lipogenesis [11]. The obesogenic effect has been found to be exerted through the activation of cannabinoid Cb1 receptors, acting in a similar way to 2-Arachidonoyl glycerol (2-AG), another endogenous ligand for the cannabinoid Cb1 receptor [12]. OEA is the second best characterized NAE, and it has been described to be a potent feeding inhibitor through the activation of PPARα receptors [13,14]. In addition, OEA regulates feeding and metabolism through the activation of the orphan receptor GP119 [15]. Its chronic administration also reduces alcohol intake and fat accumulation in the liver by activating PPARα receptors and oxidative mechanisms [14,16]. Finally, OEA attenuates neuroinflammation associated with bacterial or toxic insults [17]. Recently, OEA has been proposed to be a fat sensor, being activated after fat intake to control fat appetite and fatty acid metabolism [18]. OEA effects are synergic with the peripheral antagonism of cannabinoid receptors, suggesting that anandamide and OEA oppose each other to control energy metabolism [19]. PEA is a compound with less defined targets. PEA displays clear analgetic, anti-inflammatory, and neuroprotective actions, and its role in metabolic regulation less defined [8]. However, because of the importance of immune system activation as a pathogenic factor in both obesity and non-alcoholic steatohepatitis, PEA actions in the context of complicated obesity might contribute to healing acting as regulatory mechanism that attenuates the deleterious actions of inflammation associated with obesity [8,20].

Despite the well-characterized physiological roles of AEA, OEA, and PEA, there are other NAEs present in plasma that might have functions that are yet to be described. Among these NAEs, Linoleoylethanolamide (LEA) is very relevant. This linoleic acid-derived NAE is present in the plasma and gut at concentrations similar to those of OEA, with its concentration being much higher than other polyunsaturated NAEs [21,22]. LEA tissue levels are regulated by the type of oil intake [23]. As a nutrient, LEA is the most abundant of all NAEs; it is present in cereals (especially wheat-based foods), coffee powder, cocoa, carrots, margarine, eggs, and legumes [5]. The mastication of these foods markedly increased the presence of free LEA in saliva in [6]. However, despite its abundance, neither the roles of LEA in normal physiology nor the effects of the exogenous administration of this compound have been characterized in full. One preclinical study demonstrated that LEA is capable of reducing feeding in rodents and that this effect is abrogated by the pharmacological blockade of Ppar-α receptor, suggesting a similar role for LEA and OEA [24]. In addition, a couple of studies in humans have indicated that its concentration is elevated in obese humans and linked to cardiovascular risk [22,25]. In addition, recent reports have identified LEA as a circulating metabolic biomarker of chronic inflammatory/autoimmune disorders, although its potential contribution to the evolution or prognosis of the disease is unknown [26].

Despite this information, there are no studies addressing the potential role of LEA as a modulator of obesity and its metabolic complications. In order to gain more insight into the biological significance of this specific NAE, the present study was designed to address the following objectives: The first aim was to confirm whether plasma LEA concentrations are elevated in overweight humans not requiring treatment. This study was complemented with an analysis of the relationship of LEA with classical parameters of metabolic syndrome such as dyslipidemia or glycemia. The second objective was to investigate whether the exogenous administration of LEA is capable of restoring the metabolic/inflammatory imbalance associated with high-fat diets in a preclinical model of diet-induced obesity.

## 2. Materials and Methods

### 2.1. Populational Study: Estimating Circulating Linoleoylethanolamide Concentrations in Normal and Overweight Subjects

There are almost no studies available in the literature that analyze the significance of circulating concentrations of LEA in plasma via comparing normal and overweight people; most existing studies center around anandamide, OEA, and PEA. In order to estimate the comparative concentration of LEA in normal and overweight (BMI(Kg/m^2^) > 25) people from a typical Mediterranean community, we addressed the measurement of NAEs in one population-based cohort, Pizarra, in the south of Spain. The study population and the design of the Pizarra survey has been extensively described previously [27,28]. We randomly selected 47 participants (27 males and 20 females). Persons were excluded from the study if they were institutionalized or had mental disorders requiring treatment or chronic inflammatory disorders requiring treatment that could affect circulating endocannabinoids. The data regarding this population can be found in Table 1. All the participants completed a clinical survey and underwent an anthropometric study, as well as providing a venous blood sample that was collected in EDTA as an anticoagulant, centrifuged at the time of study, and had its plasma separated and frozen at −80 °C until later analysis. The project was approved by the ethics committee of Regional University Hospital in Malaga (see Institutional Board Statement at the end of the work).

### 2.2. Quantification of Acylethanolamides in Plasma and Liver

The analysis of acylethanolamides in the plasma and liver was performed by using a HPLC-MS method that has been previously described elsewhere [29]. The following acylethanolamides were quantified: PEA, stearoyl Ethanolamide (SEA), OEA, Palmitoleoylethanolamide (POEA), AEA, (LEA), Docosahexaenoylethanolamide (DHEA), Di-homo-γ-linolenylethanolamide (DGLEA), and Docosatetraenoylethanolamide (DEA). Briefly, aliquots of 0.5 mL of human plasma or liver homogenates were transferred to 12-mL glass tubes, spiked with deuterated internal standards, diluted with 0.1 M ammonium acetate buffer (pH 4.0), and extracted using a tert-butyl methyl ether. The dry organic extracts were reconstituted in 100 μL of a mixture of water/acetonitrile (10:90, *v*/*v*) with 0.1 percent formic acid (*v*/*v*) and transferred to HPLC vials. Twenty microliters were injected into the LC/MS-MS system. An Agilent 6410 triple quadrupole (Agilent Technologies, Wilmington, DE, USA) equipped with a 1200 series binary pump, a column oven, and a cooled auto-sampler (4 °C) was used. Chromatographic separation was carried out using an ACQUITY UPLC C18-CSH column (3.1 × 100 mm, 1.8-μm particle size) (Waters, Yvelines Cedex, France), which was then maintained at 40 °C with a mobile phase flow rate of 0.4 mL/min. The composition of the mobile phase was as follows: A: 0.1 percent (*v*/*v*) formic acid in water; B: 0.1 percent (*v*/*v*) formic acid in acetonitrile. Quantification was performed via isotope dilution. The deuterated internal standards were obtained from Cayman Chemical (Ann Arbor, MI, USA), and the solvents used were obtained from Merck (Darmstadt, Germany). MRM transitions and an example of LC/MS the chromatograms can be found in the Supplementary Materials.

### 2.3. Animals and Diets

Feeding studies and experiments related to standard diet (STD) and diet-induced obesity, induced by a high-fat palatable diet (HFD) were performed on 32 Male Sprague Dawley rats (Charles River) weighing 260–360 g at the beginning of the experiments. The animals were housed in pairs under a 12 h light/dark cycle (lights off 21:00 h) in a room with controlled temperature (20–22 °C) and humidity (45–55%) conditions. Unless otherwise indicated, the animals were given water and rat chow pellets (STD) (4RF18, Mucedola, Settimo Milanese, Italy) until the start of the diet-induced obesity procedure. All experiments were performed in accordance with the European directive 2010/63/EU governing animal welfare and protection, which is acknowledged by Italian Legislative Decree n. 116, 27 January 1992. The diet-induced obese rats were randomly divided into two groups with comparable mean body weight (no significant difference). The first group (N = 16) was fed with standard laboratory chow ad libitum (2.6 kcal/g); the second group (N = 16), was fed ad libitum with a high-fat diet palatable daily prepared diet (see Supplementary Table S1 for food components and nutritional facts) ad libitum (5.3 Kcal/g). This diet was maintained for 90 days until the rats fed with a HFD showed obesity and/or insulin resistance (see Supplementary Figure S1). Body weight and food intake for all groups were recorded daily during the study.

### 2.4. Glucose Tolerance Test

To test glucose tolerance, both the STD and HFD rats were deprived of food for 18 h prior to the procedure. The animals were administered an i.p. glucose overload of 2 g/kg body weight. Tail blood samples were collected at 0, 5, 10, 15, 30, 60, and 120 min after glucose administration. Glucose was determined using a standard glucose oxidase method (see Supplementary Figure S1).

### 2.5. Drugs and Chronic Treatment

LEA was synthesized at the Instituto de Química Médica (Madrid, Spain). LEA was dissolved in a vehicle composed of 5% Tween 80 (Sigma, St. Louis, MO, USA) diluted in 0.9% saline solution. Drugs were injected i.p. at a dose of 10 mg/Kg of body weight in a volume of 1 mL/kg of body weight for 14 days.

### 2.6. Sample Collection

At the end of chronic treatment and the administration of the two diets, the rats were sacrificed via decapitation 2 h after the last administration. Blood samples were collected (EDTA-2Na tubes) and centrifuged (1000× *g* for 10 min at 4 °C). The plasma samples were frozen at −80 °C for biochemical and hormonal analyses. Their livers were dissected and frozen in several independent pieces at −80 °C until protein expression or NAE analyses were performed.

### 2.7. Measurement of Metabolites, Hepatic Enzymes, Insulin, and Inflammatory Mediators in Plasma

The following metabolites were measured in plasma: glucose, triglycerides, total cholesterol, HDL, bilirubin, urea, uric acid, creatinine, and the hepatic enzymes GPT and GOT. They were analyzed using commercial kits according to the manufacturer’s instruction in a Hitachi 737 Automatic Analyzer (Hitachi, Tokyo, Japan). VLDL and LDL were determined via the modification of Friedewald equation78: VLDL = TG/5; LDL = [(TChol/1.19) + (TG/1.9) − (HDL/1.1) − 38]. The plasma levels of insulin, Interleukin 6 (Il-6), and Tumor necrosis factor alpha (Tnf-α) were measured using the following commercial rat kits: rat insulin ELISA kit (EZRMI-13K Millipore, St. Louis, MO, USA), Rat Il-6 ELISA Kit (KRC006 Novex, by Life technologies, Carlsbad, CA, USA), and Tnf-α Kit (KRC3011 Novex, by Life technologies, USA).

### 2.8. Hepatic Lipid Extraction and Fat Content

Fat extraction was performed via organic solvent-based extraction. Briefly, total lipids from frozen liver samples were extracted with chloroform/methanol (2:1, *v*/*v*) and butylated hydroxytoluene (0.025%, *w*/*v*). The lower phase containing lipids was separated after 2 centrifugation steps (2800× *g*, 4 °C for 10 min) and dried by nitrogen. The liver fat content was expressed as a percentage of the tissue weight.

The liver samples were analyzed for fat depots via oil red O staining. The frozen samples were cut into 30 µm-thick sections using a sliding microtome (Leica SM200R, Wetzlar, Germany) and fixed with 10% formal calcium. The sections were washed with distilled water and rinsed with 60% isopropanol. Then, the sections were stained with freshly prepared oil red O (Sigma, St Louis, MO, USA) working solution for 20 min (Oil red O stock stain: 0.5% of oil red O in isopropanol; Oil red working solution: 30 mL of the stock stain and 20 mL of distilled water). The sections were rinsed with 60% isopropanol, counterstained with Mayer’s hematoxylin, rinsed with tap water, and mounted in aqueous media.

### 2.9. Protein Extraction and Western Blot Analysis

Proteins from the tissue portions of the liver samples were extracted on ice using a lysis buffer (40 mM Tris-HCl pH 7.4, 200 mM NaCl, 4% Triton ×100, 20 mM EDTA pH 8) containing a proteinase and phosphatase inhibitor cocktail (5 mg/mL leupeptin, 100 mM NaF, 1 mM sodium orthovanadate, 5 μg/mL aprotinin, 1 μg/mL pepstatin A, 10 μg/mL trypsin inhibitor, 1 mM phenylmethylsulfonyl fluoride, 0.75 μL/mL protease and phosphatase inhibitor cocktail). Protein concentration was determined via the Bradford protein assay. Equivalent amounts of protein extract (30 μg) were separated in 10% SDS-PAGE gels (NuPAGE™ Novex™ 10% Bis-Tris Midi Protein Gels, #WG1201A; Thermo Fisher, Waltham, MA, USA) using a SDS running buffer (NuPAGE^®^ MOPS SDS Running Buffer, #NP0001; Thermo Fisher) and then electroblotted onto 0.2 μm nitrocellulose membranes (Bio-Rad, Hercules, CA, USA). The membranes were blocked in TBS-T (50 mM Tris-HCl pH 7.6, 200 mM NaCl and 0.1% Tween 20) with 2% albumin fraction V from bovine serum (BSA; Roche, Basel, Switzerland) for 1 h at room temperature. Specific proteins were detected by incubation overnight at 4 °C and raised against the following rabbit antibodies: t-Acc (#3676S; Cell Signaling Technology, Leiden, The Netherlands), p-Acc (#3661S; Cell Signaling), Fas (#3180S; Cell Signaling Technology), Acox (#ab39072; Abcam, Boston, MA, USA), Scd1 (#ab19862; Abcam), Faah (#101600; Cayman, USA), Nape-Pld (#10306; Cayman), Cb1 (#ab23703; Abcam), Cb2 (#ab3561; Abcam)), Gpr55 (#ab228735; Abcam), Cpt1a (#ab128568; Abcam), Nfkβ (#8242S Cell Signaling), Ikβ (#ab32518, Abcam), Ikkβ (#8943S, Cell Signaling), iNos (#PA1-036, Thermo Fisher Scientific), Cox-2 (#12282, Cell Signaling), Ppar-α (#ab8934; Abcam), Ucp1(#ab209483; Abcam), and Ucp2 (#ab67241; Abcam). The mouse anti-γ-adaptin (#610385; BD Biosciences, Madrid, Spain) was used as a reference protein. All antibodies were diluted 1:1000 in TBS-T and 2% BSA. After extensive washing in TBS containing 1% Tween 20, HRP-conjugated anti-rabbit or anti-mouse IgG (H + L) secondary antibodies (Promega, Madison, WI, USA) (diluted 1:10,000) were added for 1 h at room temperature. After enhanced chemiluminescence detection (Santa Cruz Biotechnologies, Dallas, TX, USA) in an Autochemi-UVP Bioimaging System, bands were quantified using ImageJ software (Rasband, W.S., ImageJ, U.S., NIH, http://imagej.nih.gov/ij, version 153 for windows, accessed on 20 July 2023). Images of all gels with either Ponceau staining or specific antibody labeling can be found in the Supplementary Materials.

### 2.10. Statistical Analysis

Data are expressed as the mean ± standard error of the mean (SEM) of at least 8 determinations per experimental group. Statistical results were obtained using the computer program GraphPad Prism version 6.01 (GraphPad Software Inc., San Diego, CA, USA). Differences were analyzed via a 1-way or 2-way ANOVA depending on the factors and kind of analysis, followed by the Bonferroni post hoc test for multiple comparisons, Student’s unpaired T test, or the Mann–Whitney U test where appropriate. A *p*-value below 0.05 was considered statistically significant.

## 3. Results

### 3.1. Population Characteristics and Plasma Concentrations of Linoleoylethanolamide in Normal and Overweight People

Table 1 presents the socio-demographic data and metabolic characteristics of the participants randomly selected from the Pizarra database. Most variables were distributed normally. However, lipid concentrations were not distributed normally in the studied population; thus, we used the Mann–Whitney U Test for pairwise comparisons. After stratification based on the criteria of the control population (Body mass index (BMI (Kg/m^2^)) < 25, n = 13) and overweight population (BMI (Kg/m^2^) > 25, n = 34), we found significant differences in BMI (with the obese population having greater BMI values (t = 7.726, df = 45, *p* < 0.001), lower plasma HDL cholesterol (Mann–Whitney U = 87, *p* < 0.05), and a higher number of plasma triglycerides (Mann–Whitney U = 88, *p* < 0.05)), indicating that part of the overweight/obese people also have dyslipidemia, a fact that was not observed in any of the lean participants.

**Figure 1:**
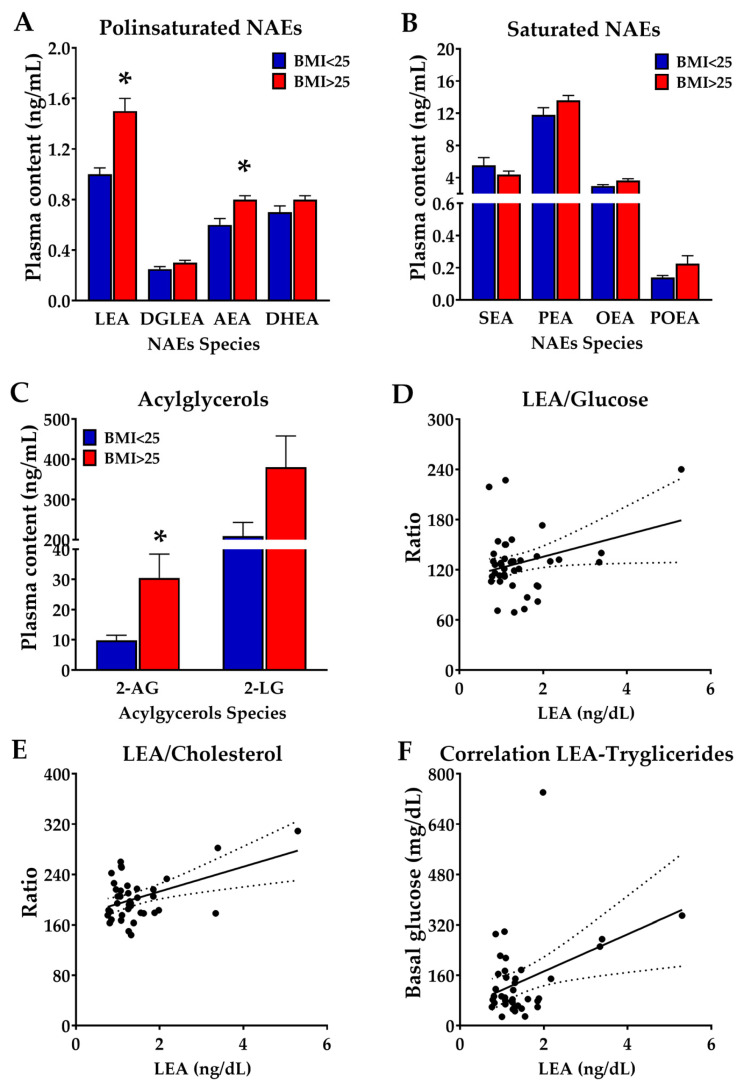
(**A**,**B**); Plasma concentrations of NAEs in a representative Mediterranean population of Spain (city of Pizarra) stratified on the basis of their body mass index in lean (BMI < 25, N = 13) versus obese (BMI > 25, N = 34) subjects. (**C**) Plasma concentration of the acylglycerols 2-Arachidonoyl glycerol (2-AG) and 2-LG in the same population. Correlation of plasma LEA concentrations with that of (**D**) glucose, (**E**) cholesterol, and (**F**) triglycerides. Dashed lines correspond to confidence intervas * *p* < 0.05 versus BMI < 25 patients (ANOVA).

In this population, we me measured the circulating concentrations of NAEs and the concentrations of two acylglycerols, namely the endocannabinoid 2-Arachidonoyl glycerol (2-AG) and the linoleic acid-containing one, 2-Linoleoylglycerol (2-LG), in the plasma. Our results are shown in Figure 1. Obese/overweight participants displayed enhanced concentrations of polyunsaturated NAEs (F (1, 176) = 8.879, *p* < 0.005), with important differences being observed between NAEs species (F (3, 176) = 46.00, *p* < 0.001). Thus, LEA was the most abundant polyunsaturated NAE, followed by AEA. Post hoc analysis revealed that both LEA and AEA concentrations were elevated in obese people with respect to the control (lean) participants (Figure 1A). Regarding saturated and mono-unsaturated NAEs, the analysis indicated important differences in the concentrations of NAEs species (F (3, 176) = 197.4; *p* < 0.001), with saturated NAEs being more abundant than monounsaturated ones. An overweight condition did not influence either the monounsaturated or saturated NAEs (F (1, 176) = 0.8416; *p* = 0.38, non-significant) (Figure 1B). The acylglycerol concentrations were different among species, with LG being more abundant than 2-AG (F (1, 88) = 18.77; *p* < 0.001). Our post hoc analysis revealed that 2-AG concentrations were higher in overweight participants (Figure 1C). Interestingly, plasma concentrations of LEA correlated with that of glucose (F (1, 40) = 4.36; *p* < 0.04), plasma cholesterol (F (1, 37) = 11; *p* < 0.005), and plasma triglycerides (F (1, 37) = 6.95; *p* = 0.01).

### 3.2. Effects of Subchronic Administration of Linoleoylethanolamide in Weight Gain and Food Intake in Animals Receiving a HFD

**Figure 2:**
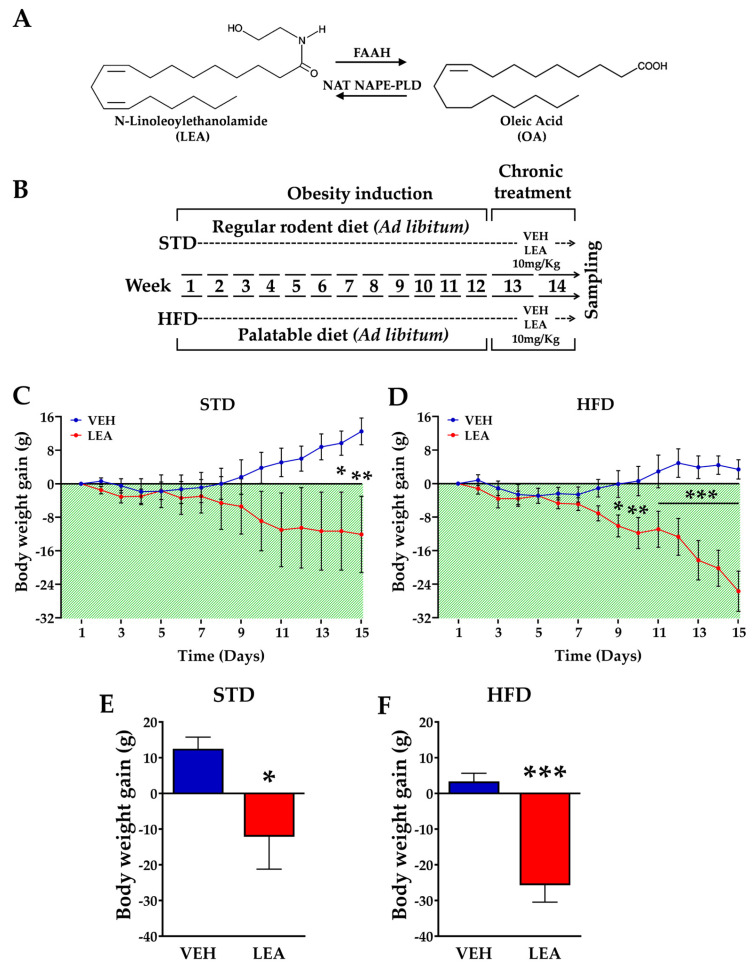
(**A**) Chemical structure of LEA and linoleic acid and the enzymes involved in their degradation (Faah) and synthesis (Nat, Nape-Pld). (**B**) Design of the study on the effects of LEA administration in eight male rats (N = 6–8 animals/group). (**C**,**D**) Daily effect of LEA treatment on weight gain in the animals of the two diets groups. (**E**,**F**) Cumulative weight gain after 15 days of treatment; * *p* < 0.05, ** *p* < 0.01, and *** *p* < 0.001 denote LEA versus VEH group (two-way ANOVA).

Exposure to HFD for 12 weeks resulted in enhanced caloric intake, weight gain, and the development of glucose intolerance when compared with animals receiving a STD (Supplementary Figure S1). Once this obese/glucose-intolerant condition was achieved, the subchronic administration of LEA for 15 days resulted in weight loss (treatment effect, F (1, 24) = 22.77; *p* < 0.001). Our post hoc analysis showed that the effect of LEA was more intense in the animals fed with the HFD (Figure 2C–F). The effect of LEA on weight gain was not immediate since it appeared after at least 7 days of treatment (F (14, 210) = 6.179; *p* < 0.001). Cumulative food intake was not altered by LEA administration in any of the two diet groups (F (1, 24) = 0.84; non-significant, Supplementary Figure S2).

### 3.3. Effects of Chronic Administration of Linoleoylethanolamide in Plasma Biochemistry

Exposure to a HFD altered all of the plasma biochemical parameters measured; namely, we observed enhanced circulation levels of glucose, insulin, triglycerides, cholesterol, urea, uric acid, creatinine, bilirubin, and the transaminases AST and ALT (see Table 2). Treatment with LEA corrected some of the effects of HFD; it lowered glucose (diet × treatment interaction F (1, 24) = 9.604; *p* < 0.005), triglycerides (diet x treatment interaction F (1, 24) = 6.07; *p* < 0.03), cholesterol (diet × treatment interaction F (1, 24) = 22.03; *p* < 0.001), uric acid (diet × treatment interaction F (1, 24) = 10.08; *p* < 0.005), creatinine (diet × treatment interaction F (1, 24) = 13.68; *p* < 0.005), bilirubin (diet × treatment interaction F (1, 24) = 4.92; *p* < 0.04), and AST (diet × treatment interaction F (1, 24) = 14.68; *p* < 0.001). LEA treatment did not affect the impact of HFD on insulin, urea, or ALT (Table 2).

### 3.4. Effects of Sucbchronic Administration of Linoleoylethanolamide in Plasma Il-6 and Tnf-α

**Figure 3:**
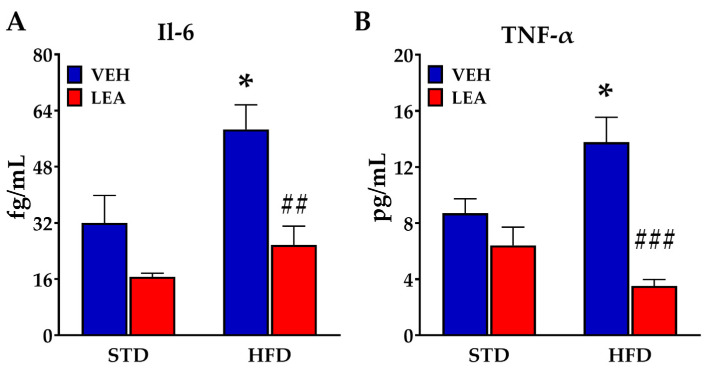
LEA treatment reversed the HFD-induced increase in the plasma concentration of Interleukin-6 (Il-6, **A**) and tumor necrosis factor alfa (Tnf-α, **B**). Data are presented as means ± standard error of the mean (N = 6–8 animals/group). * *p* < 0.05 different versus vehicle group; ## *p* < 0.01 and ### *p* < 0.01 different versus VEH-treated group of the same diet (two-way ANOVA).

In order to analyze whether LEA treatment can reverse inflammation associated with a HFD, the concentrations of both Il-6 and Tnf-α in plasma were measured (See Figure 3). The HFD resulted in a clear increase in both Il-6 (F (1, 24) = 17.04; *p* < 0.001, Figure 3A) and Tnf-α (F (1, 16) = 30.94; *p* < 0.001; Figure 3B). This pro-inflammatory effect of the HFD was reversed by the administration of LEA, which reduced both Il-6 (F (1, 24) = 9.35; *p* < 0.01) and Tnf-α (F (1, 16) = 12.48; *p* < 0.005). In order to analyze the impact of these anti-inflammatory actions of LEA, we analyzed the signaling cascade of the Tnf-α activated NfκB signaling cascade in the liver (see Supplementary Figure S3). LEA was able to counteract the small increase in Nfκβ associated with a high-fat diet (diet x treatment interaction, F (1, 23) = 5.2; *p* < 0.04). Interestingly, the LEA treatment was also associated with an increase in the expression of the kinase Ikkβ (treatment effect, F (1, 23) = 15.3; *p* < 0.001). An enhanced expression of Ikkβ facilitates the degradation of the NFκβ inhibitor Ikβ, thus resulting in a release of Nfκβ. Although this effect should facilitate the expression of pro-inflammatory genes such as cytokines (Il-6) or iNos/Cox enzymes, that response was not observed after LEA treatment, suggesting that this NAE abrogates NFκB-mediated responses via a mechanism that is yet to be discovered.

### 3.5. Effects of Chronic Administration of Linoleoylethanolamide on Liver Steatosis and the Expression of Lipid Metabolism-Related Enzymes in the Liver

**Figure 4:**
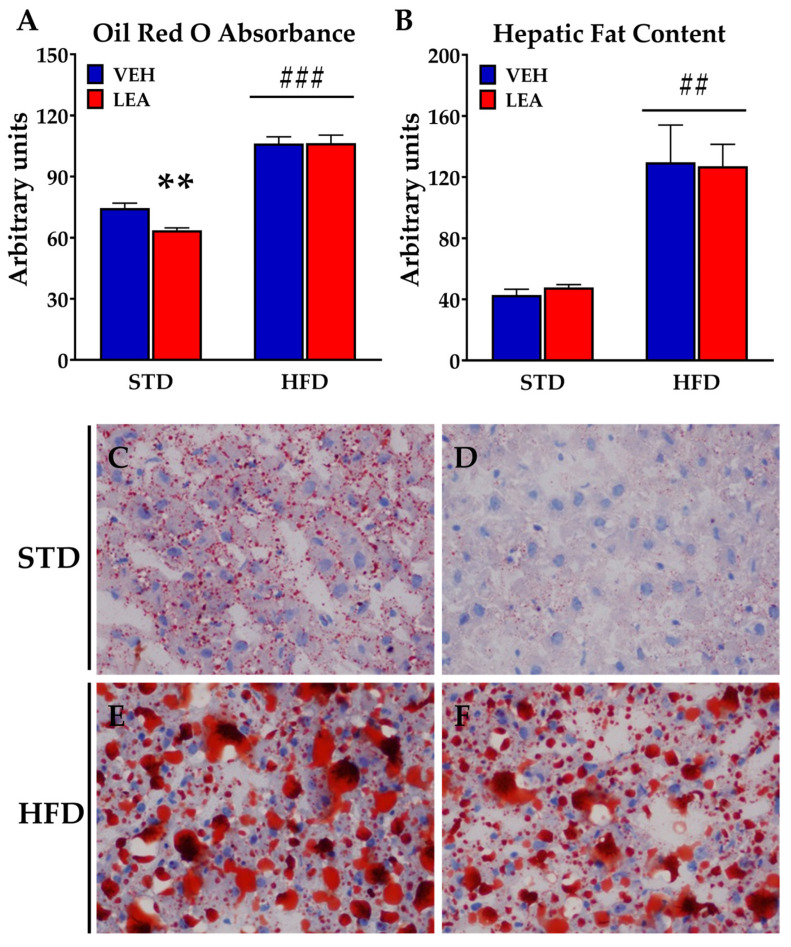
LEA treatment did not reverse the liver steatosis associated with HFD exposure in male rats. (**A**) Fat content measured using oil red staining; (**B**) fat content measured using organic extraction; (**C**) oil red staining of a liver section from a STD–VEH-treated animal; (**D**) oil red staining of a liver section from a STD–LEA-treated animal; (**E**) oil red staining of a liver section from a HFD–VEH-treated animal; (**F**) oil red staining of a liver section from a HFD–LEA-treated animal. Data are presented as means ± standard error of the mean (N = 6–8 animals/group). ** *p* < 0.01 versus STD-fed animals treated with vehicle; ## *p* < 0.01 and ### *p* < 0.001 versus STD-fed animals treated with LEA (two-way ANOVA).

**Figure 5:**
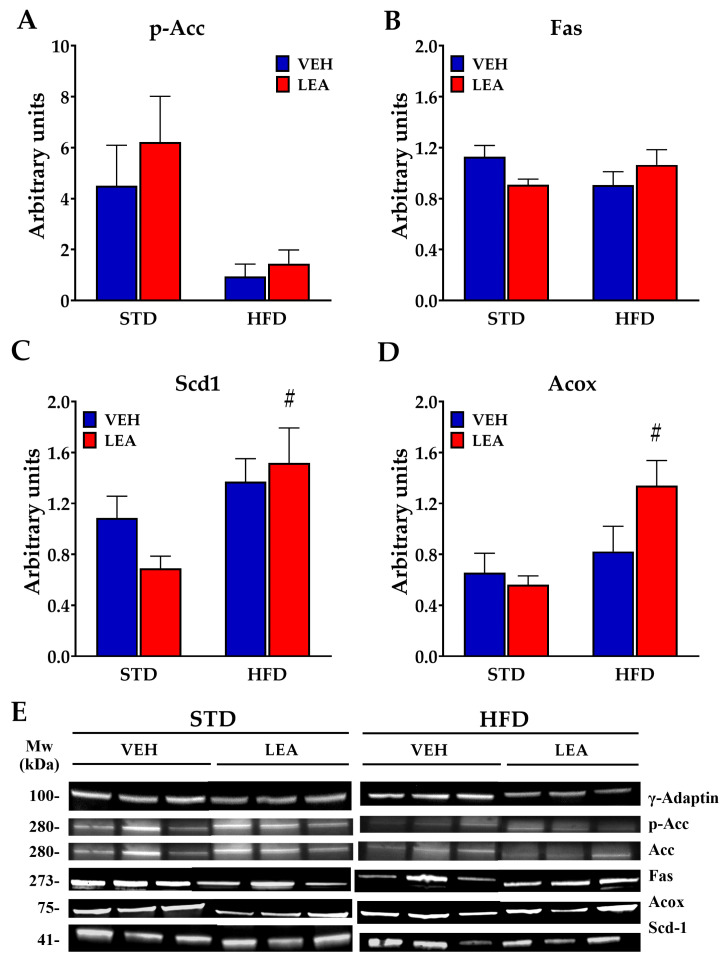
LEA treatment did not modify the alterations in the lipogenic pathway in the liver of male rats exposed to a HFD, but it did activate peroxisomal oxidation. (**A**) The HFD activated Acetyl-CoA-Carboxylase (Acc) by removing inhibitory phosphorylation; (**B**) neither HFD nor LEA modified fatty acid synthase (Fas); (**C**) exposure to the HFD increased the activity of the Stearoyl-CoA-desaturase 1 (Scd1) enzyme; (**D**) LEA treatment increased the expression of peroxisomal acyl-CoA-oxidase (Acox). (**E**) Representative blots immunostained for each of the proteins tested. Data are presented as means ± standard error of the mean (N = 6 animals/group). # *p* < 0.01 versus STD-fed animals treated with LEA (two-way ANOVA).

Since NAEs are relevant modulators of lipid biosynthesis and degradation and are dysregulated in obesity, we evaluated the impact of LEA administration on obese animals. Exposure to a HFD resulted in a marked increase in liver steatosis, measured via both oil-red staining (F (1, 24) = 153.2; *p* < 0.001) and organic fat extraction (F (1, 23) = 23.18; *p* < 0.001). LEA treatment did not reduce HFD-induced obesity, according to both of the two methods (treatment effect using oil red O staining (F (1, 34) = 3.2; *p* = 0.08); treatment effect using organic extraction (F (1, 23) = 0.1; n.s.)), but our post hoc analysis revealed a significant decrease in fat depots in the animals in the STD group treated with LEA (observed using red oil staining) (Figure 4). The lack of effects on lipid accumulation in the liver is supported by the small impact of LEA treatment on the main enzymes related to lipogenesis (Figure 5). HFD exposure resulted in the activation of the lipogenesis mediated by acetyl-CoA-carboxylase (Acc), as reflected by the removal of inhibitory phosphorylation in HFD animals (diet effect, F (1, 20) = 11.13; *p* < 0.005, Figure 5A), without affecting the total amount of the enzyme Acc (see supplementary information on western blots). LEA treatment did not affect this reduced phosphorylation of Acc (treatment effect F (1, 20) = 0.80; n.s.).

However, the fatty acid synthase complex Fas was not modulated by either HFD exposure or LEA treatment (Figure 5B). Stearoyl-CoA desaturase-1 (Scd1), an obesity-linked enzyme essential for the synthesis of mono/polyunsaturated fatty acids, was enhanced by the HFD (diet effect, F (1, 20) = 8.47; *p* < 0.01, Figure 5C) but, once again, it was not affected by LEA treatment. Regarding lipid oxidation, we found that while LEA promoted the expression of Acox, facilitating peroxisomal lipid oxidation (F (1, 20) = 8.45; *p* < 0.01, Figure 5D), it reduced the expression of the HFD-enhanced Cpt1α, suggesting a reduction in mitochondrial β-oxidation (Supplementary Figure S4).

**Figure 6:**
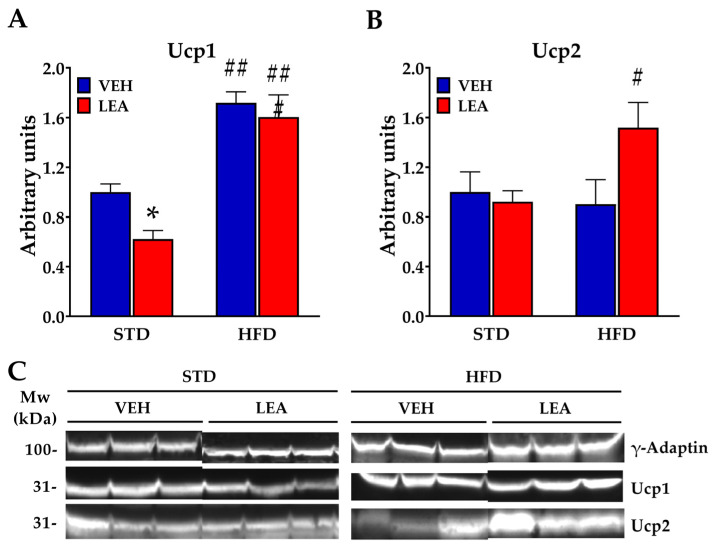
LEA treatment modified the expression of uncoupling proteins in the liver. (**A**) HFD exposure enhanced the expression of Uncopling protein 1 (Ucp1), while LEA treatment in STD-exposed animals reduced its expression; (**B**) LEA treatment in HFD-exposed animals increased the expression of Uncopling protein 2 (Ucp2). (**C**) Representative blots immunostained for each of the proteins tested. Data are presented as means ± standard error of the mean (N = 6 animals/group). * *p* < 0.05 versus STD-fed animals treated with vehicle; # *p* < 0.05, ## *p* < 0.01, and ### *p* < 0.001 versus STD-fed animals treated with LEA (two-way ANOVA).

Finally, in order to complete our analysis of lipid metabolism in the liver, we analyzed the expression of mitochondrial uncoupling proteins (UCPs) 1 and 2. Ucp1 was enhanced by the HFD (diet effect, F (1, 20) = 58.3; *p* < 0.001, Figure 6A), and LEA treatment only reduced it in STD-exposed animals (treatment effect, F (1, 20) = 4.85; *p* < 0.05, Figure 6B). The expression pattern of Ucp2 in the liver was different from that of Ucp1 (Figure 6B). Ucp2 was not affected by diet, but a marked increase on its expression was observed after LEA treatment (Diet x treatment interaction, F (1, 20) = 4.4; *p* < 0.05).

### 3.6. Effects of Chronic Administration of Linoleoylethanolamide on the Expression of Acylethanolamide-Related Enzymes in the Liver

**Figure 7:**
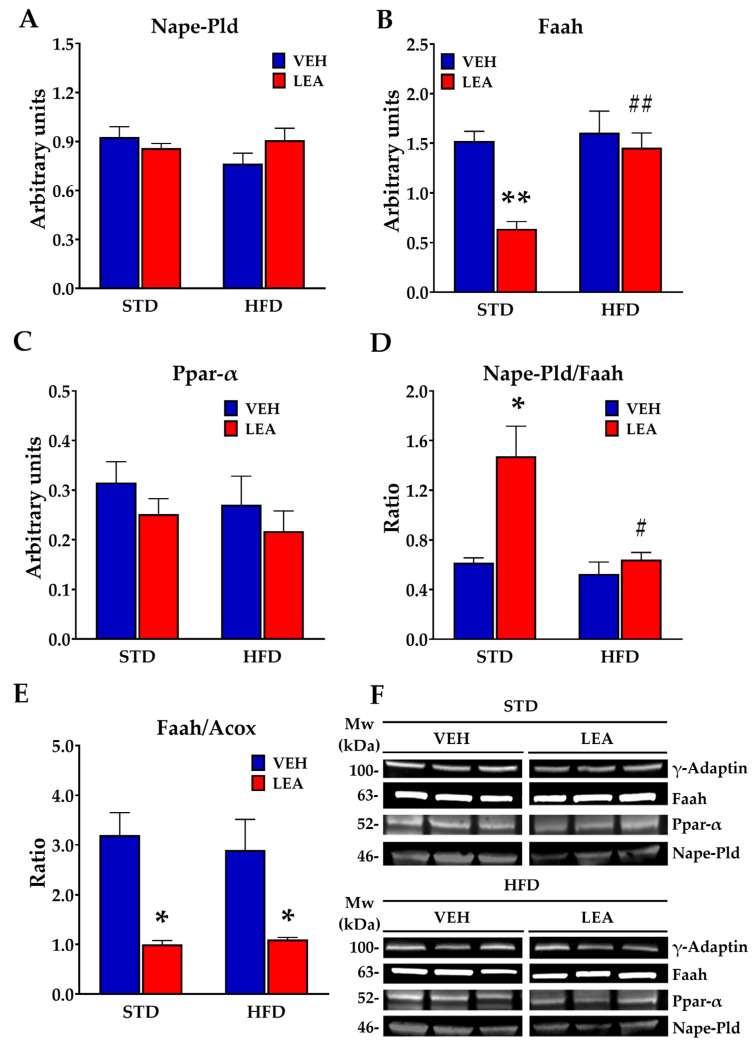
Effects of HFD exposure and/or LEA treatment on NAE signaling machinery. (**A**) Neither HFD nor LEA modified the NAE-releasing enzyme N-acyl phosphatidylethanolamine-specific phospholipase D (Nape-Pld); (**B**) LEA treatment reduced the expression of Faah, the main NAE-degrading enzyme, in the STD-fed animals; (**C**) neither HFD nor LEA modified the NAE receptor Ppar-α; (**D**) treatment with LEA markedly enhanced the production/degradation ratio of NAEs in the STD-fed animals; (**E**) LEA treatment markedly reduced the Faah/Acox ratio in the liver of animals independently of the diet; (**F**) representative blots immunostained for each of the proteins tested. Data are presented as means ± standard error of the mean (N = 6 animals/group). * *p* < 0.05, ** *p* < 0.01 versus STD-fed animals treated with vehicle; # *p* < 0.05 and ## *p* < 0.01 versus STD-fed animals treated with LEA (two-way ANOVA).

Since endocannabinoid and NAEs have been involved in lipogenesis and lipo-oxidation [19], we evaluated the impact of the subchronic administration of LEA on the protein expression of the NAE’s signaling machinery (Figure 7).

Regarding the main NAE-releasing enzyme, Nape-Pld, neither exposure to the HFD (F (1, 20) = 0.93; n.s.) nor the treatment with LEA (F (1, 20) = 0.39; n.s.) modified the protein expression of this enzyme (Figure 7A). However, both exposure to the HFD (F (1, 20) = 9.68; *p* < 0.005) and LEA treatment (F (1, 20) = 12.84; *p* < 0.001) modified the expression of the main NAE-degrading enzyme Faah. A significant diet x treatment interaction (F (1, 20) = 6.45; *p* < 0.02) revealed that LEA reduced Faah expression in the STD-fed animals, an effect that was not observed in HFD-exposed animals. Interestingly, Faah expression correlated with Acyl-CoA-oxidase (Acox) expression (F (1, 22) = 8.6; *p* < 0.01, Supplementary Figure S5), and the Faah/Acox ratio was markedly modulated by LEA treatment (treatment effect, F (1, 20) = 22.93; *p* < 0.0001, Figure 7E). Regarding the expression of NAE receptors, we observed that the expression of Ppar-α receptors (the main receptor for saturated and mono-unsaturated NAEs) was not affected by either diet or LEA treatment (Figure 7C). HFD exposure increased pro-lipogenic cannabinoid Cb1 (diet effect, F (1, 20) = 8.34; *p* < 0.01) receptors in the liver and decreased anti-inflammatory cannabinoid Cb2 receptors (diet effect, F (1, 20) = 8.47; *p* < 0.01). However, LEA treatment did not affect the expression of both cannabinoid receptors. (Supplementary Figure S6). Finally, neither HFD nor LEA treatment modified the expression of G protein-coupled receptor 55 (Gpr55), a putative cannabinoid receptor activated by Lysophosphatidyl inositol.

### 3.7. Effects of Chronic Administration of Linoleoylethanolamide on Acylethanolamide Contents in the Liver

**Figure 8:**
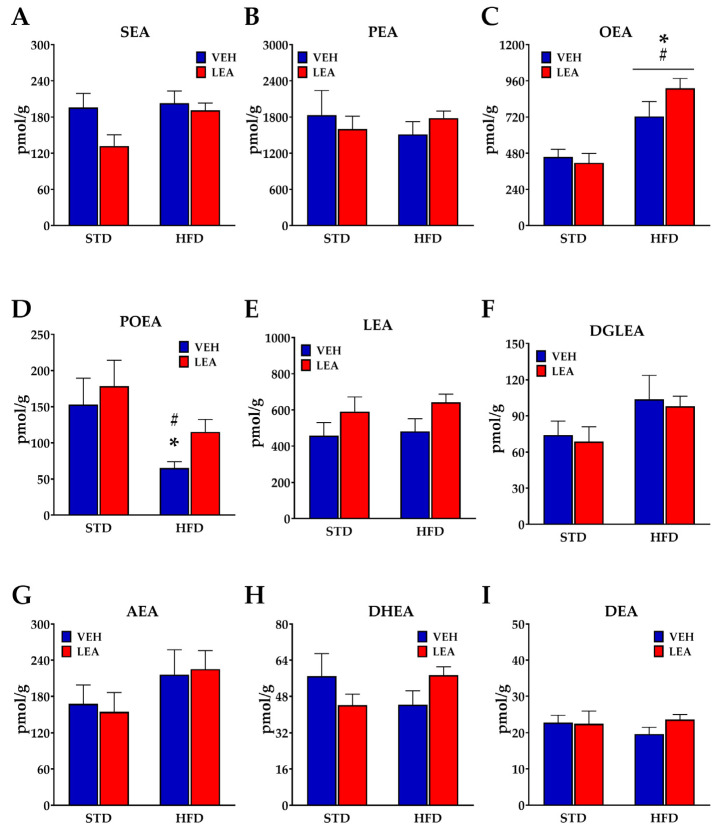
Effects of either HFD exposure or LEA treatment on the content of NAEs in the liver of male rats. (**A**) Stearoylethanolamide, SEA; (**B**) palmitoylethanolamide, PEA; (**C**) oleoylethanolamide, OEA; (**D**) palmitoleoylethanolamide, POEA; (**E**) linoleoylethanolamide, LEA; (**F**) di-homo-γ- linolenylethanolamide, DGLEA; (**G**) arachidonoylethanolamide, AEA; (**H**) docosatetraenoylethanolamide, DEA and (**I**) docosahexaenoylethanolamide, DHEA. Data are presented as means ± standard error of the mean of the six to eight determinations per group (N = 6–8). * *p* < 0.05 versus STD-fed animals treated with vehicle; # *p* < 0.05 versus STD-fed animals treated with LEA (two-way ANOVA).

Since LEA has been proposed to modify NAE degradation by interfering with Faah, the main NAE-degrading enzyme [30], we monitored the concentrations of NAEs in the liver tissues (Figure 8). Interestingly, the only net LEA treatment-derived effect observed was an increase in LEA contents in the liver (F (1, 23) = 4.6; *p* < 0.05, Figure 8E). The remaining effects observed were derived from exposure to the HFD. Thus, the concentrations of either SEA (F (1, 23) = 4.3; *p* < 0.05, Figure 8A), OEA (F (1, 23) = 25.7; *p* < 0.0001, Figure 8C) and DGLEA (F (1, 23) = 4.5; *p* < 0.05, Figure 8F) in the liver were reduced, whereas that of POEA (F (1, 23) = 8.61; *p* < 0.05, Figure 8D) was decreased after HFD exposure. The case of OEA was particularly significant, doubling the concentrations of OEA. The remaining NAEs were not modified by HFD exposure or the subchronic administration of LEA.

## 4. Discussion

The present study confirms the findings of previous studies that suggest that LEA is a relevant NAE implicated in both the metabolic and the inflammatory imbalances associated with obesity [22,25]. While our study on a standard Mediterranean population indicated that plasma concentrations of LEA are associated with obesity and dyslipidemia, the preclinical study regarding diet-induced obesity revealed that the chronic administration of LEA resulted in the normalization of dyslipidemia, an improvement in liver function, and a reduction in inflammatory markers associated with long-term exposure to high-fat diets. Taken together, the data suggest that LEA is potentially part of the defensive response of the organism to the homeostatic challenge imposed by complicated obesity. This idea is supported by a recent study in psychotic patients, where a decrease in LEA was associated with long-term antipsychotic treatment and considered a significant biomarker of antipsychotic-induced obesity and metabolic syndrome [31]. Further studies are needed to identify the potential significance of the LEA’s response to the metabolic shifts imposed by nutrients, lifestyle choices, and drugs. LEA is the most abundant NAE in both foods and edible oils made up of seeds [5,23]. Plants accumulate LEA in seeds to provide a source of signals for controlling seedling development [32]. Its presence in most of the vegetable foods consumed by humans (cereals, legumes, seed oils, etc.) means that we have relevant sources of LEA, the role of which in nutrition needs to be fully studied. In addition, LEA has been linked to sport endurance [33] through its association with vascular non-inflammatory molecule-1 (Vanin-1), a pantetheinase necessary for the synthesis of coenzyme A, an essential molecule for lipid biosynthesis and energy metabolism [34]. To which extent this association reflects an important role for LEA on controlling lipid metabolism needs to be fully understood.

To gain insight into the pharmacology of LEA and its impact on metabolism, we investigated the pharmacological actions of LEA in a preclinical model of diet-induced obesity. Firstly, it is important to highlight that LEA has a particular profile with respect to cannabinoid/non-cannabinoid signaling. On one hand, it has been described to be a competitive inhibitor of Faah, slowing the degradation of AEA [30]. This might result in an indirect cannabimimetic action that might promote feeding and lipogenesis. On the other hand, it has been proposed to activate Ppar-α receptors, resulting in satiety and lipid oxidation profiles [35]. Although both possibilities might have biological significance, the truth is that we lack conclusive information on the real mechanism of action of this NAE. Taking this particular pharmacological profile of LEA in consideration, the present study contains relevant information that might help to clarify the biological significance of LEA. Concerning food intake and body weight gain, our data indicate that LEA does not generate a long-term reduction in food intake, while it does block, or even reduce, body weight gain. Although LEA is usually described as an anorectic NAE, a careful analysis of published data reveals that this effect is restricted in time, lasting around one hour [24]. Thus, it is feasible that the animals treated chronically with LEA did not show an overt reduction in feeding behavior. The reduction in body weight gain might thus be related to a metabolic adaption towards oxidative metabolism, something that we will discuss below. In addition, LEA administration reduced cholesterol and triglycerides, but not insulinemia in the diet-induced obese animals. This profile of LEA is thus intermediate in between cannabimimetic AEA [12] (which enhances feeding, body weight gain, and lipogenesis while promoting insulin resistance through the activation of cannabinoid receptors such as Cb1) and that of the non-cannabinoid NAEs OEA and POEA, two monounsaturated NAEs that reduce feeding and body weight gain; lower insulin, cholesterol, and triglycerides; and act through the activation of Ppar-α receptors [32].

With respect to the reduction in body weight gain observed in the HFD-exposed animals treated with LEA, the results obtained are contradictory and, again, different from those reported for OEA. We did not find that the LEA-induced modulation of the lipogenic pathway activated by HFD, as described for OEA or POEA [32]. In fact, fat depots in the liver remained intact, while the Acc and Scd1 lipogenic enzymes remained increased despite LEA administration. Apparently, this should have enhanced fat deports and body weight gain, even more so if we consider the reduction in the export of triglycerides observed after LEA treatment. However, what we observed was a non relevant reduction in fat in the liver of STD-fed animals and no effect on the HFD-fed ones, associated with a small reduction in body weight gain. A potential explanation for this might center around the LEA treatment-induced expression of Acox, a peroxisomal enzyme that promotes the non-mitochondrial β-oxidation of very long fatty acids [36]. The activity of this enzyme serves as a sensor for regulating lipolysis [37]. However, its overactivity might result in cell damage through the increased production of reactive oxygen species. This potential damage is counteracted by the enhanced expression of Ucp2 in obese animals treated with LEA. Ucp2, located in the inner mitochondrial membrane, lowers mitochondrial membrane potential and dissipates metabolic energy, preventing the damage associated with oxidative stress accumulation [38]. In fact, this cytoprotective role for LEA can be extended to the cytokine-mediated inflammatory response; LEA was also capable of reducing the circulating levels of both Il-6 and Tnf-α, thus reducing potential cytokine-mediated hepatic damage. In fact, plasma biomarkers of hepatic lesions (for instance, levels of AST transaminase, bilirubin, or uric acid) were normalized after LEA treatment. The mechanism of this anti-inflammatory action remains to be conclusively determined, although potential interaction with the Nfkβ signaling pathway appeared to occur in the liver of the LEA-treated obese animals.

The effects described herein seem to be directly dependent on LEA and not on other NAEs that are dysregulated by LEA treatment. Despite the potential interference of LEA with the NAE degradation pathway because of its interference with Faah activity [30], we did not find changes in other NAE contents in the liver after two weeks of LEA treatment. LEA was the only NAE affected after LEA administration, as reflected by the increased levels measured. The remaining NAEs only changed as a result of HFD exposure, further supporting the notion that the precursors and release of NAEs depend on the fatty acids received through the diet [4]. In addition, LEA treatment did not affect Nape-Pld expression or Ppar--α receptors, but it did modulate Faah expression in the STD-fed animals. In this regard, it is important to note that we observed a relevant association of Faah with Acox: the tissue contents of both proteins are directly related, suggesting that Faah activity is enhanced together with non-mitochondrial β-oxidation. This association was found to be modulated by LEA, and its significance is yet to be studied.

## 5. Conclusions

Overall, the present data suggest that LEA is a relevant bioactive NAE whose plasma levels are enhanced in obesity associated with dyslipidemia as part of the defensive response of the organism to the challenges associated with a high-fat diet/obesity. The exogenous administration of LEA is capable of normalizing the metabolic and inflammatory alterations associated with long-term exposure to high-fat diets. However, subchronic treatment with LEA was not capable of reducing food intake or liver steatosis. LEA could potentially be used as a supplement for improving hepatic function in complicated obesity; it can be considered as an adjunct therapy, although it has limitations imposed by liver steatosis and excessive body weight. Future studies aiming to estimate the normal range of LEA consumption regarding different diets should be conducted to understand whether there is a dietary need for this supplement.

## Figures and Tables

**Table 1:** Characteristics of a sample of mediterranean population (Pizarra, Málaga, Spain). Data are presented as means ± standard deviation (SD) of selected antropometric and metabolic variables. ** *p* < 0.01 Student’s *T* test. # *p* < 0.05 Mann–Whitney U test. BMI, Body mass index (Kg/m^2^); HBA1c, glycosylated hemoglobin; n.s. non-significant.

Parameter	BMI (Kg/m^2^) < 25 (N = 13)	BMI (Kg/m^2^) > 25 (N = 34)	*p* Value
Age	42.6 ± 3.0	44.5 ± 3.4	n.s.
BMI	23.7 ± 1.3	28.5 ± 2.1 **	<0.0001
Basal Glucose (mg/dL)	126.0 ± 18.5	113.7 ± 57.3	n.s.
% HBA1c	5.2 ± 0.2	5.4 ± 0.4	n.s.
Cholesterol (mg/dL)	185.4 ± 21.8	208.9 ± 37.8	n.s.
LDL Cholesterol (mg/dL)	116.9 ± 20.1	130.8 ± 39.1	n.s.
HDL Cholesterol (mg/dL)	52.9 ± 7.9	45.1 ± 16.4 #	<0.05
Triglycerides (mg/dL)	77.7 ± 27.2	166.1 ± 143.1 #	<0.05

**Table 2:** Plasma biochemical parameters in both STD-fed and HFD-fed animals treated daily for 15 days with LEA (10 mg/kg). Data are expressed as means ± SEM (N = 6–8 animals/group) and were analyzed via a two-way ANOVA (diet and treatment and Bonferroni post hoc test. * *p* < 0.05, ** *p* < 0.01, and *** *p* < 0.001 indicate the existence of significant differences when compared with VEH group. # *p* < 0.05 statistically different from the STD–LEA group.

Plasma Metabolites	Standard Diet (STD)		High-Fat Diet (HFD)	
	VEH	LEA	VEH	LEA
**Glucose (mg/dL)**	164.00 ± 8.45	157.33 ± 7.34	311.80 ± 42.3 ***	168.57 ± 4.49 #
**Insulin (ng/mL)**	3.94 ±0.55	3.28 ± 0.57	6.92 ± 1.51 *	9.10 ± 1.48 *
**Triglycerides (mg/dL)**	173.00 ± 7.68	146.57 ± 13.29	414.3 ± 93.7 **	150.29 ± 12.26 #
**Cholesterol (mg/dL)**	68.38 ± 4.80	75.2 ± 8.88	211.00 ± 35.35 ***	48.28 ± 3.76 #
**Urea (mg/dL)**	16.86 ± 1.40	14.00 ± 1.29	72.92 ± 3.29 ***	62.00 ± 13.66 **
**Uric acid (mg/dL)**	2.28 ± 0.34	1.63 ± 0.24	9.38 ± 1.95 ***	2.27 ± 0.33 #
**Creatinine (mg/dL)**	0.75 ± 0.09	0.69 ± 0.20	1.74 ± 0.12 **	0.71 ± 0.05 #
**Bilirubin (mg/dL)**	0.18 ± 0.02	0.21 ± 0.03	0.99 ± 0.31 **	0.25 ± 0.13
**AST (UI)**	150.57 ± 19.91	138.00 ± 27.06	553.00 ± 41.37 **	288.33 ± 34.16 #
**ALT (UI)**	53.14 ± 9.66	85.00 ± 3.56	206.33 ± 28.93 **	192.17 ± 27.64 *

## Data Availability

Not applicable.

## Supplementary Materials

The following supporting information can be downloaded at: https://www.mdpi.com/article/10.3390/nu15204448/s1.
