# Supplementary material for: Administration of Linoleoylethanolamide Reduced Weight Gain, Dyslipidemia, and Inflammation Associated with High-Fat-Diet-Induced Obesity
Source: Nutrients. 2023 Oct 20;15(20):4448. doi: 10.3390/nu15204448 (PMC10609991; doi:10.3390/nu15204448)
Supplement: Supplementary file 1 [file nutrients-15-04448-s001.zip › nutrients-2603830-supplementary.pdf]

Supplementary Materials

ADMINISTRATION OF LINOLEOYLETHANOLAMIDEREDUCED WEIGHT GAIN, DYSLIPAEMIA AND INFLAMMATION ASSOCIATED WITH HIGH FAT DIET-INDUCED OBESITY

Rubén Tovar[1†], Marialuisa de Ceglia[1†], Massimo Ubaldi[2], Miguel Rodríguez-Pozo[1], Laura Soverchia[2], Carlo Cifani[2], Gema Rojo[3], Ana Gavito[1], Laura Hernandez-Folgado[4], Nadine Jagerovic[4], Roberto Ciccocioppo[2] , Elena Baixeras[1], Fernando Rodríguez de Fonseca[1,5,6*]and Juan Decara[1,*]

1. DIET CHARACTERISTICS
2. EFFECTS ON FEEDING AND GLUCOSE TOLERANCE
3. EFFECTS ON INFLAMMATORY MARKERS
4. EFFECTS ON MARKERS OF LIPID OXIDATION
5. NAEs BIOANALYSIS: DETAILS
6. PONCEAU RED-STAINED WESTERN BLOT MEMBRANES

SUPPLEMENTARY TABLE S1: COMPOSITION OF CAFETERÍA DIET

**HFD**	**Description**	**Amount**	**Kcal**	**Carbohydrate**	**Protein**	FAT		% COMPOSITION ON FINAL MIXTURE					
**Palatable Diet**	**(Italian Food)**	**(%)**	**(/g)**	**(g/100g)**	**(g/100g)**	**Saturated**	**Unsaturated**	**Protein**	**Carbohydrate**	**Saturated**	**Unsaturated**	**Salt**	**Fiber**
Mortadella	*Mortadella Italiana*	10	3,21	0,50	15,5	8	17	**1,55**	**0,05**	**0,8**	**1,7**	**0,23**	0
Rusk	*Crostini*	8	5,53	48,00	8,1	5,4	6,6	**0,648**	**3,84**	**0,432**	**0,528**	**0,208**	0,112
Cookie with cream	*Macine con panna*	18	5,44	68,00	6	5,1	12,9	**1,08**	**12,24**	**0,918**	**2,322**	**0,1386**	0,27
Chocolate muffin	*Muffin con ciocolatto*	22	4,56	53,00	5,1	3,1	13,9	**1,122**	**11,66**	**0,682**	**3,058**	**0,22**	0,726
Cheese	*Parmeggiano Reggiano*	14	4,38	0.0	29	17	11	**4,06**	**0**	**2,38**	**1,54**	**0,224**	0
Snack	*Patatine al formaggio*	16	5,35	59,00	6,8	3,3	21,7	**1,088**	**9,44**	**0,528**	**3,472**	**0,368**	0,512
Flavoured lard	*Lardo* (50% mixed with 4RF18 Diet)	12	9,04	25,10	12	34	53	**1,44**	**0**	**4,08**	**6,36**	**0,78**	0,42
total		100	37,51	253,6	82,5	75,9	136,1	10,988	37,23	9,82	18,98	2,1686	2,04

COMPOSITION OF CAFETERÍA DIET

**HFD**	**Description**	**Amount**	**Kcal**	**Carbohydrate**	**Protein**	FAT		% COMPOSITION ON FINAL MIXTURE					
**Palatable Diet**	**(Italian Food)**	**(%)**	**(/g)**	**(g/100g)**	**(g/100g)**	**Saturated**	**Unsaturated**	**Protein**	**Carbohydrate**	**Saturated**	**Unsaturated**	**Salt**	**Fiber**
Mortadella	*Mortadella Italiana*	10	3,21	0,50	15,5	8	17	**1,55**	**0,05**	**0,8**	**1,7**	**0,23**	0
Rusk	*Crostini*	8	5,53	48,00	8,1	5,4	6,6	**0,648**	**3,84**	**0,432**	**0,528**	**0,208**	0,112
Cookie with cream	*Macine con panna*	18	5,44	68,00	6	5,1	12,9	**1,08**	**12,24**	**0,918**	**2,322**	**0,1386**	0,27
Chocolate muffin	*Muffin con ciocolatto*	22	4,56	53,00	5,1	3,1	13,9	**1,122**	**11,66**	**0,682**	**3,058**	**0,22**	0,726
Cheese	*Parmeggiano Reggiano*	14	4,38	0.0	29	17	11	**4,06**	**0**	**2,38**	**1,54**	**0,224**	0
Snack	*Patatine al formaggio*	16	5,35	59,00	6,8	3,3	21,7	**1,088**	**9,44**	**0,528**	**3,472**	**0,368**	0,512
Flavoured lard	*Lardo* (50% mixed with 4RF18 Diet)	12	9,04	25,10	12	34	53	**1,44**	**0**	**4,08**	**6,36**	**0,78**	0,42
total		100	37,51	253,6	82,5	75,9	136,1	10,988	37,23	9,82	18,98	2,1686	2,04

FIGURE S1

A

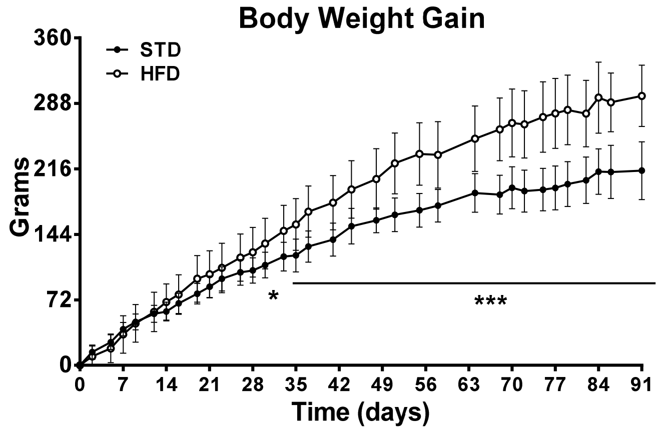

B

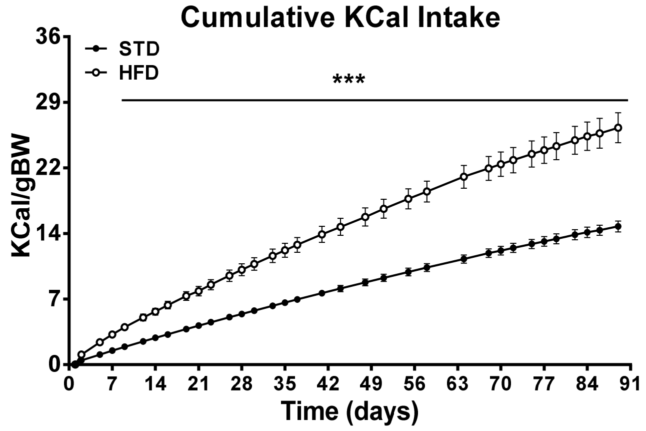

C

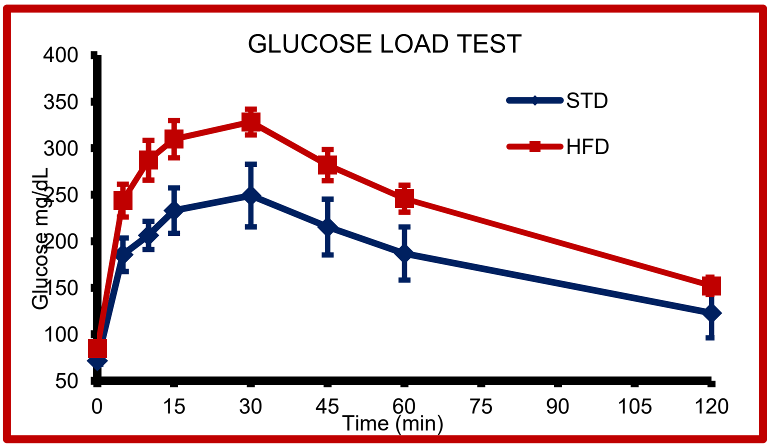

Supplementary Figure S1. Body weight gain; (A) and cumulative KCal intake; (B) during STD and HFD feeding for 90 days starting LEA/VEH treatment. ; (C) Effect of HFD in plasma glucose level after a glucose load. Values are expressed as mean ± SD (n=28-30 animals/group). Two-way ANOVA and Bonferroni post-hoc test: (*) P< 0.05 and (***) P< 0.001 vs STD group.

FIGURE S2

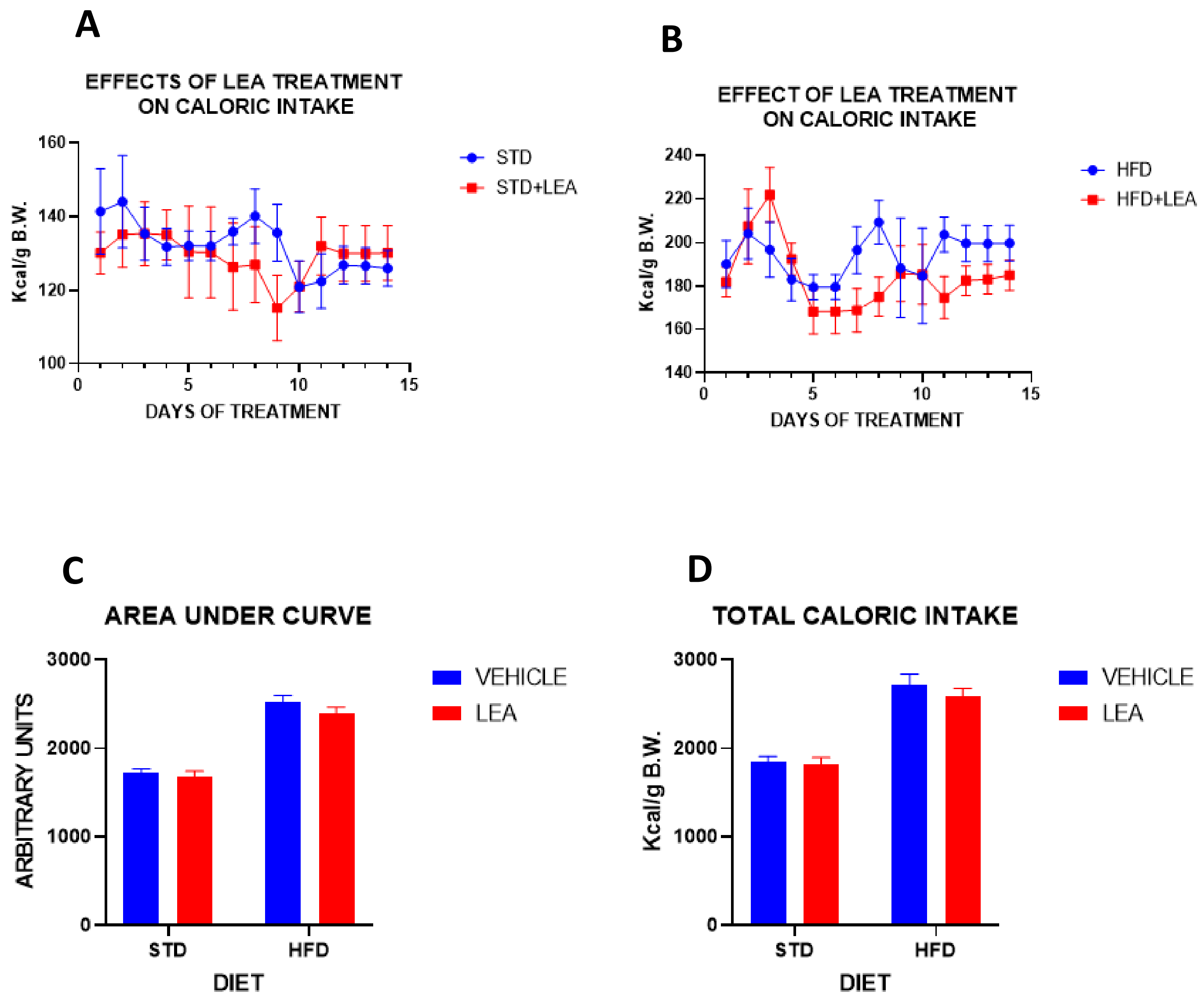

Supplementary Figure S2. (A) daily caloric intake on animals fed standard diet and (B) daily caloric intake on animals fed a high fat diet along the 15 days of LEA treatmet. (C) and (D) display the area under the curve of groups depicted on A and B, and the cummulative caloric intake respectively. Data suggests that repeated LEA administration does not affect total food intake in any of the two diet groups. Values are expressed as mean ± SD (n=7-8 animals/group). Two-way ANOVA and Bonferroni post-hoc test.

FIGURE S3

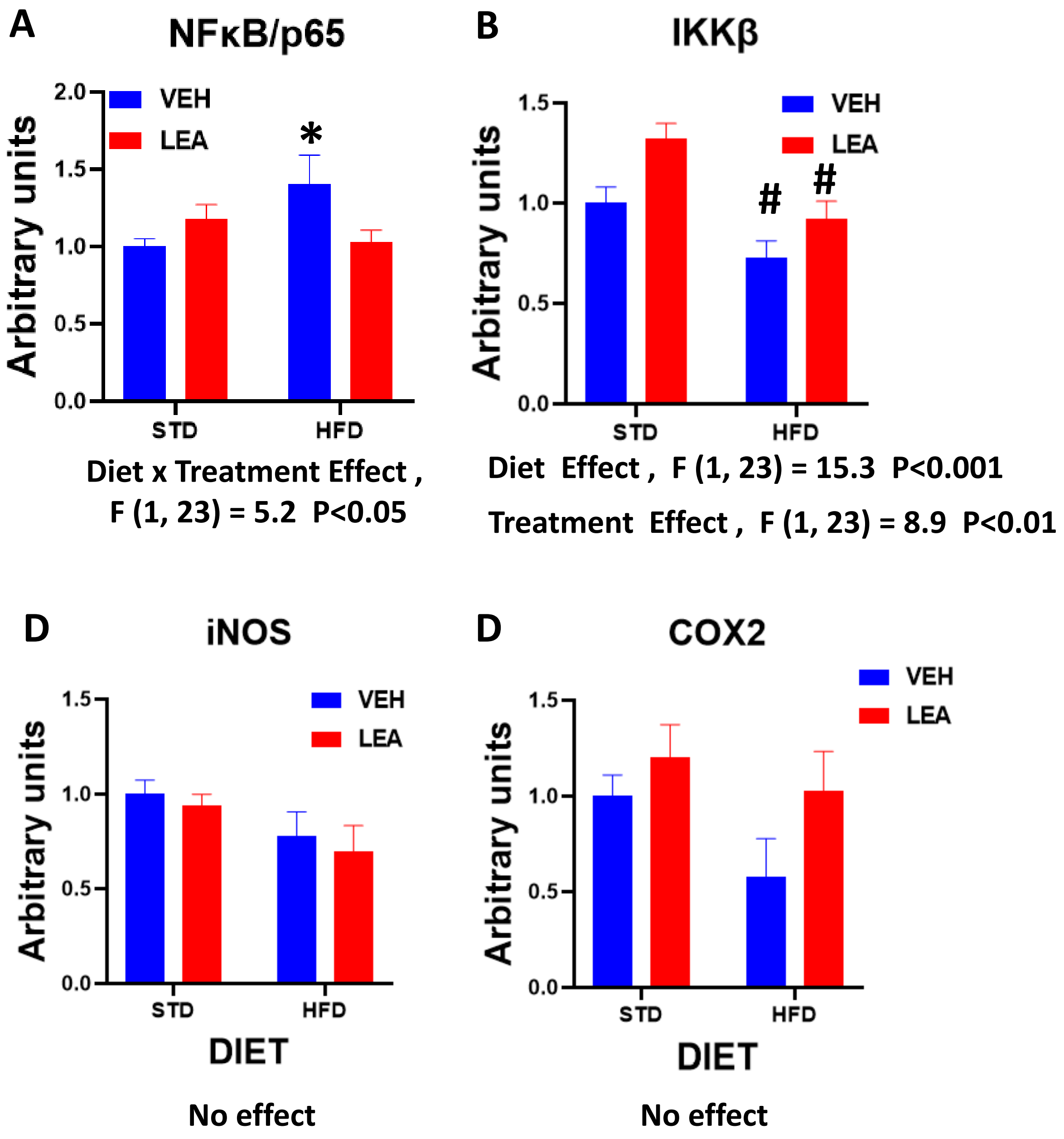

Supplementary Figure S3. Effects of HFD and LEA treatment on inflammatory signaling , measured by western blot: : (**A**) NFκB/p65 protein; (**B**) inhibitor of nuclear factor kappa B kinase subunit beta IKKb ; (**C**) inducible nitric oxide synthase, inos(**D**) Ciclooxigenase-2, COX2. Values are expressed as mean ± S.E.M. (n=5-8 animals/group). Two-way ANOVA and Bonferroni post-hoc test: (*) P< 0.05 versus STD-VEH group, and # P< 0.01 vs STD-LEA group.

FIGURE S4

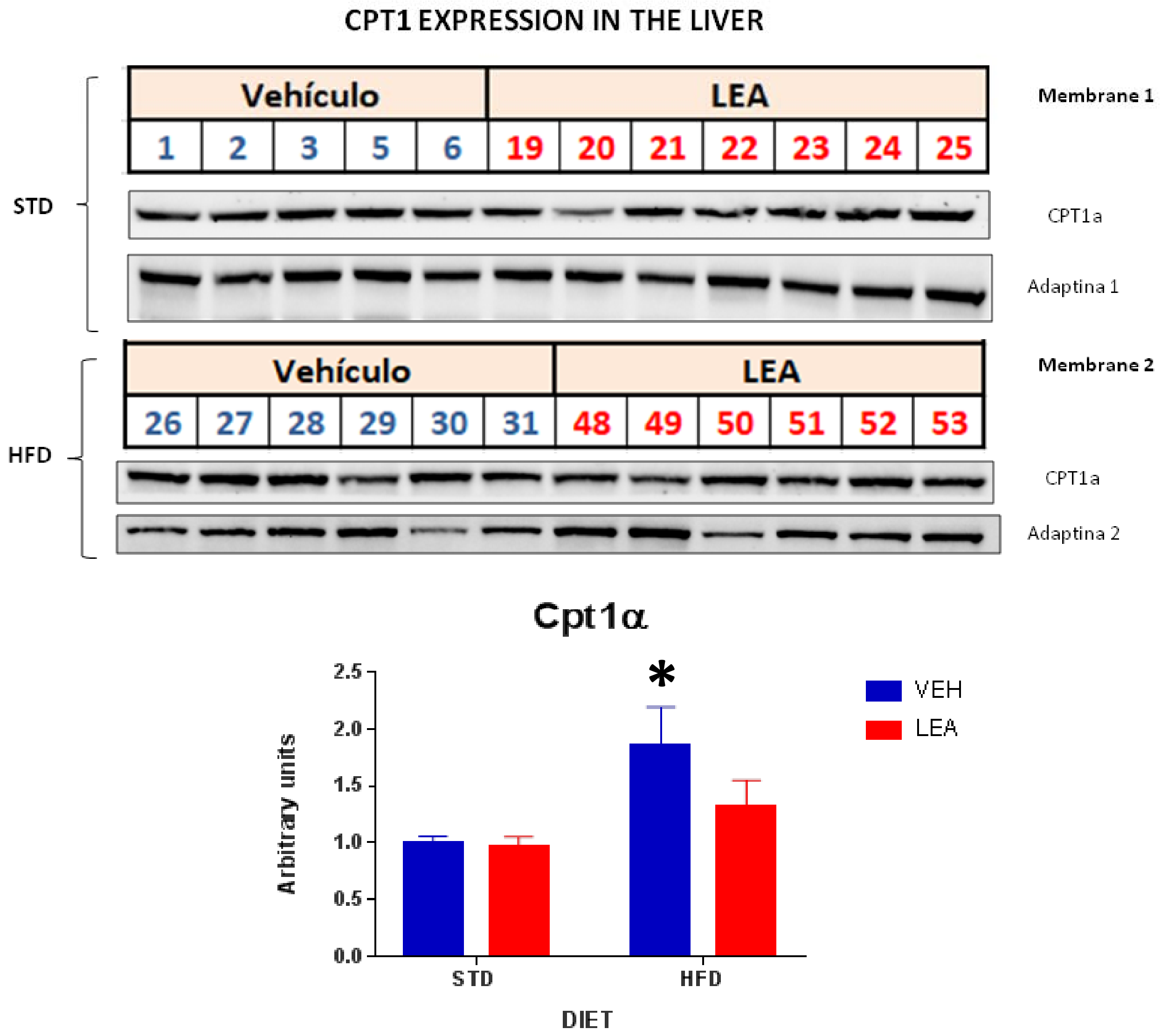

Supplementary Figure S4. Effects of HFD and LEA treatment on carnitine palmitoyltransferase I alpha (Cpt1α) expression in the liver, as measured by western blot. Values are expressed as mean ± S.E.M. (n=6-8 animals/group). Two-way ANOVA and Bonferroni post-hoc test: (*) P< 0.05 versus STD-VEH group.

FIGURE S5

CORRELATION FAAH-ACOX

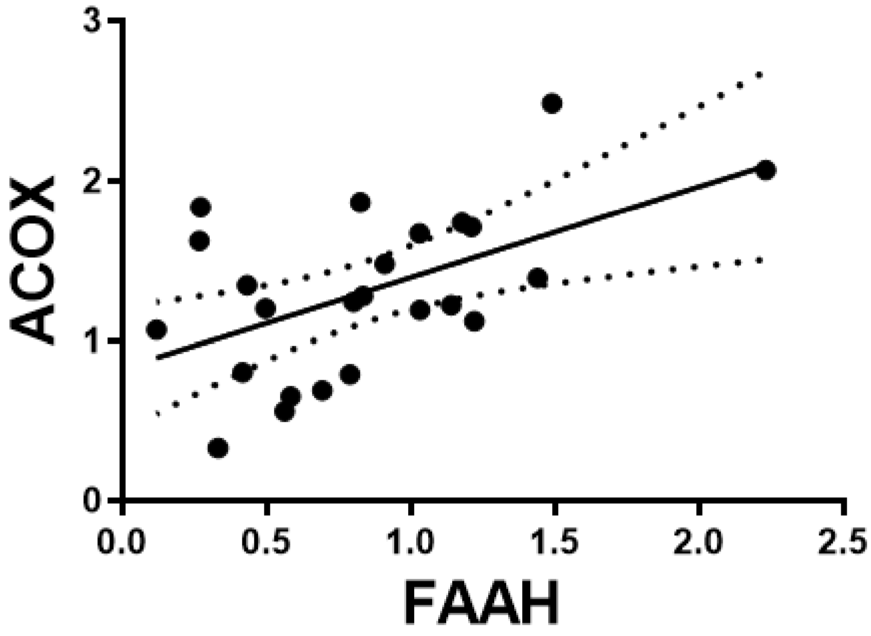

95% Confidence Intervals	
Slope	0,1670 to 0,9709
Y-intercept	0,4370 to 1,215
X-intercept	-7,015 to -0,4669
**Goodness of Fit**	
R squared	0,2815
Sy.x	0,4480
F	8,618
DFn, DFd	1, 22
P value	0,0077

Supplementary Figure S5. Correlation in between the expression of acetyl-CoA-oxidase (ACOX) and fatty acid amidohydrolase (FAAH) expression, measured by western blots. The data from the four experimental groups were included for this correlation analysis.

FIGURE S6

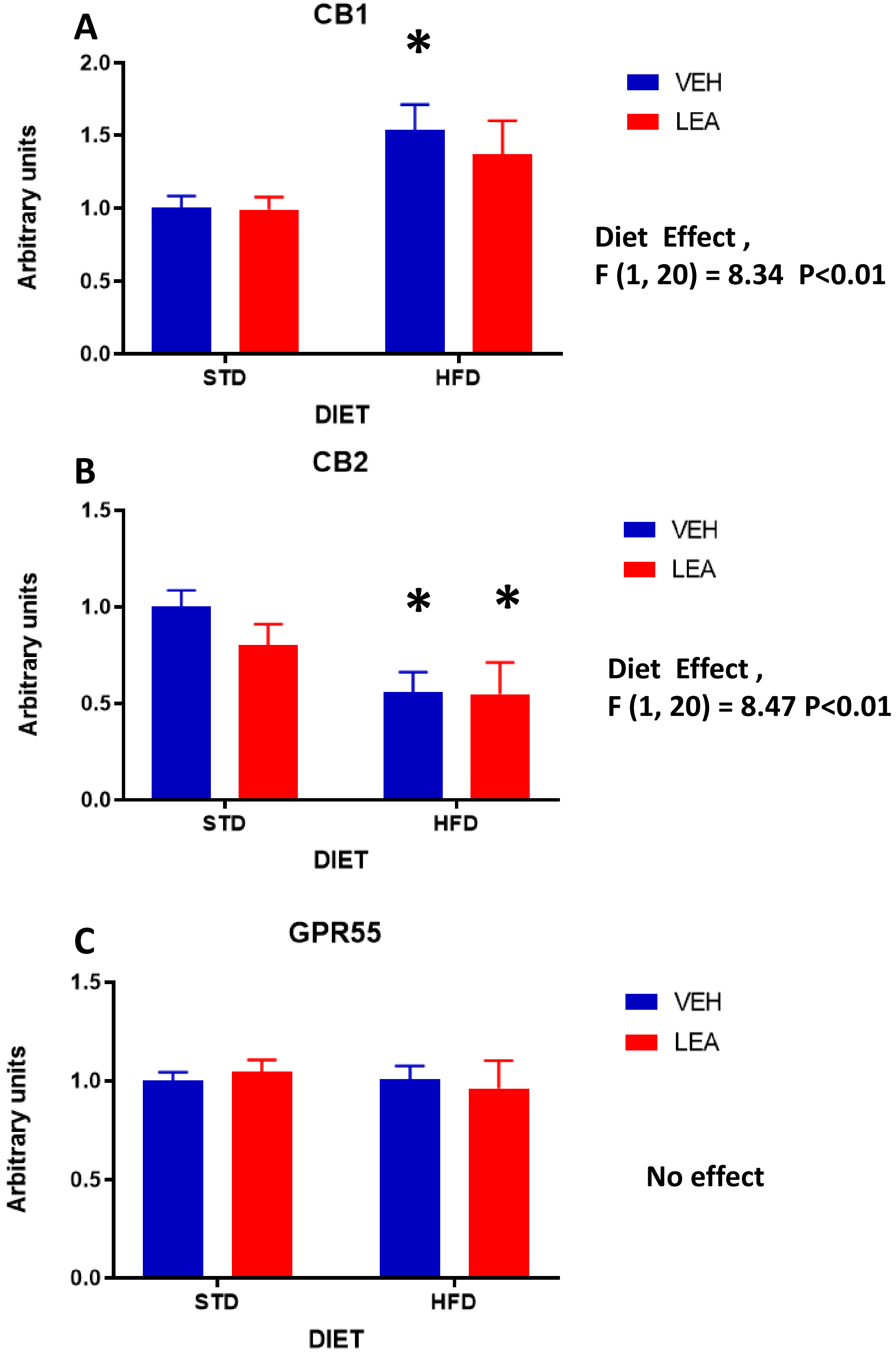

Supplementary Figure S6. Effects of HFD and LEA treatment on cannabinoid receptors expression, measured by western blot: (**A**) Cannabinoid CB1 receptors; (**B**) Cannabinoid CB2 receptors; (**C**) GPR55 receptors. Values are expressed as mean ± S.E.M. (n=6-8 animals/group). Two-way ANOVA and Bonferroni post-hoc test: (*) P< 0.05 versus STD-VEH group.

Analysis of NAEs. The various N-acylethanolamides were identified and quantified with the following MRM signals In positive electrospray mode using a Waters Acquity UPLC® system with a Xevo TQ-Smicro Mass Spectrometry detector: Authentic standards were used for the identification and the quantification was done by isotopic dilution using the response of isotopic analogues as described in a previous publication: *Pastor A, Farré M, Fitó M, Fernandez-Aranda F, de la Torre R (2014) Analysis of ECs and related compounds in plasma: artifactual isomerization and ex vivo enzymatic generation of 2-MGs. J Lipid Res 55:966-977.*

Supplementary Table S2.
Bioanalytical m/z ratios
For MS detection of NAEs.

**Analyte**	**Quantification signal**	**Identification signal**
AEA	m/z 348>62	m/z 348>44
DEA	m/z 376>62	m/z 376>44
DGLEA	m/z 350>62	m/z 350>44
DHEA	m/z 372>62	m/z 372>44
LEA	m/z 324>62	m/z 324>44
OEA	m/z 326>62	m/z 326>44
PEA	m/z 300>62	m/z 300>44
POEA	m/z 298>62	m/z 298>44
SEA	m/z 328>62	m/z 328>44

FIGURE S7

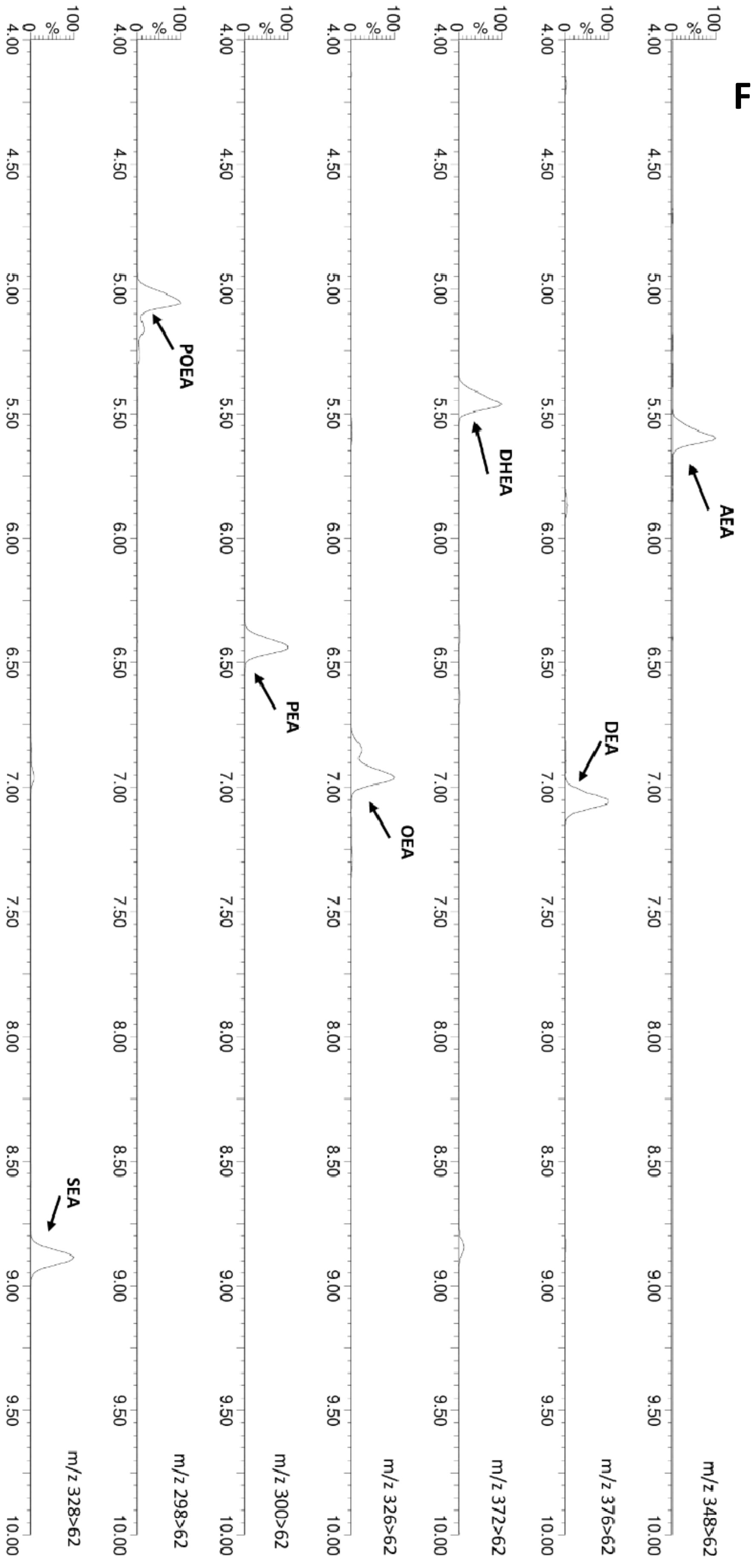

Supplementary Figure S7. Example of a typical MS chromatogram showing some of the NAES analyzed in the present study

Original Western Blot images:
Ponceau Red stained membranes

Whole membranes from Gel 1,2 .3and 4. Red

Ponceau S stain

GEL 1

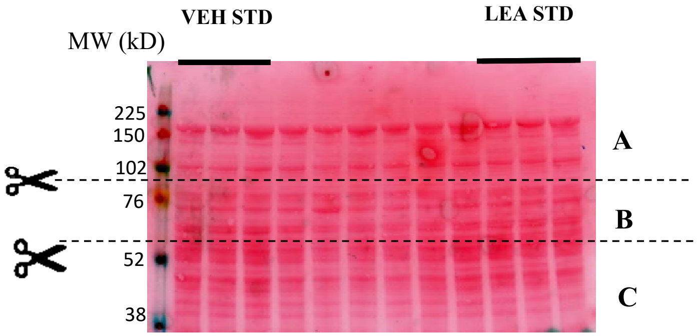

(≈ 41 Kd) Scd11
(≈ 273 kD) Fas
(≈ 75 kD) Acox 1
(≈ 63 kD) Faah
(≈ 105 kD) / γ-Adaptin

Ponceau S stain

GEL 3

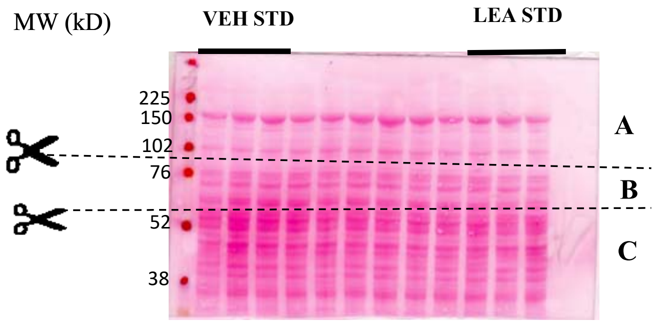

(≈ 41 Kd) Scd11
(≈ 273 kD) Fas
(≈ 75 kD) Acox 1
(≈ 63 kD) Faah
(≈ 105 kD) / γ-Adaptin

Ponceau S stain

GEL 2

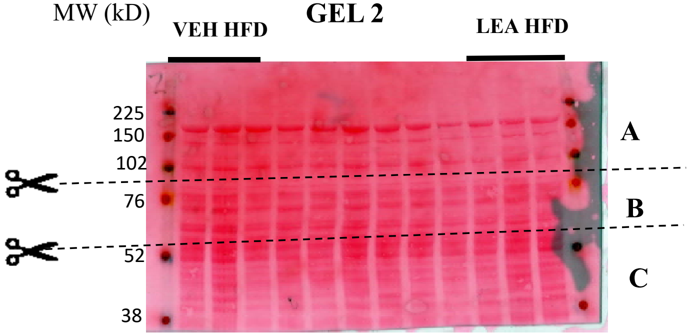

(≈ 41 Kd) Scd11
(≈ 273 kD) Fas
(≈ 75 kD) Acox 1
(≈ 63 kD) Faah
(≈ 105 kD) / γ-Adaptin

Ponceau S stain

GEL 4

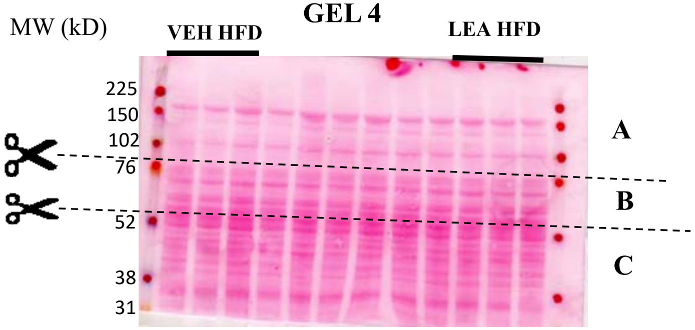

(≈ 41 Kd) Scd11
(≈ 273 kD) Fas
(≈ 75 kD) Acox 1
(≈ 63 kD) Faah
(≈ 105 kD) / γ-Adaptin

Whole membranes from Gel 1,2 .3and 4. Red

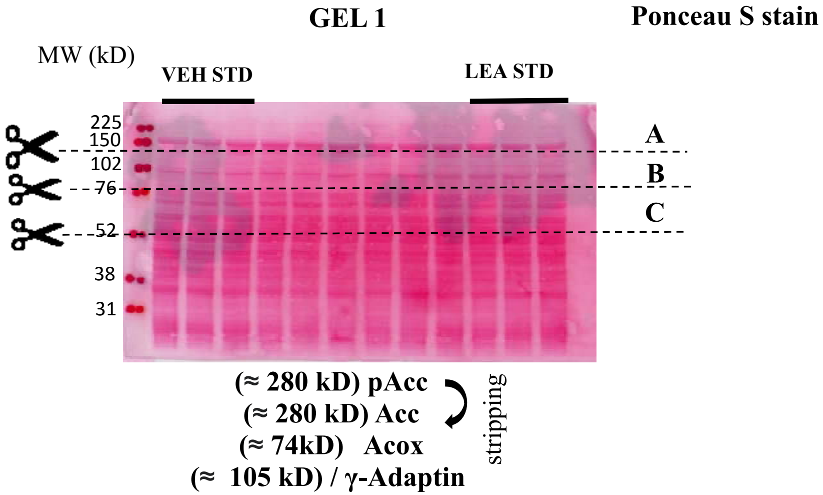

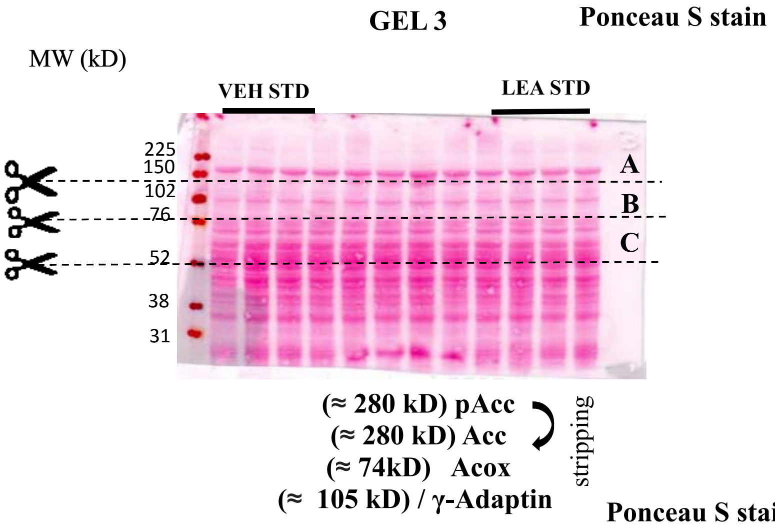

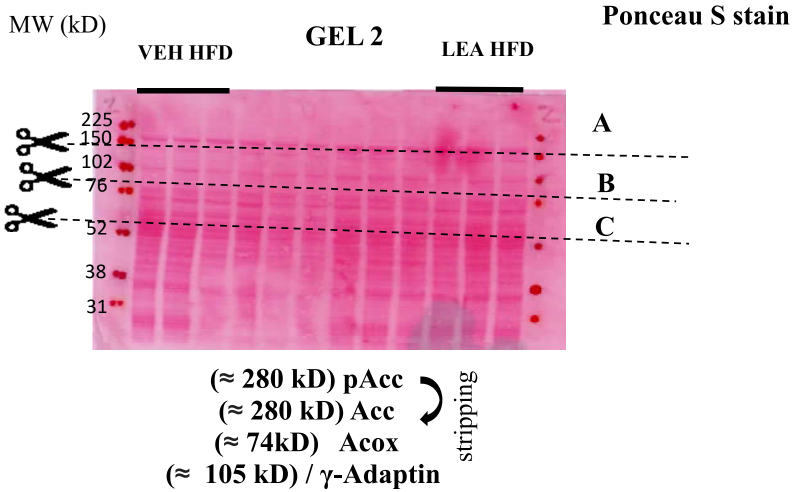

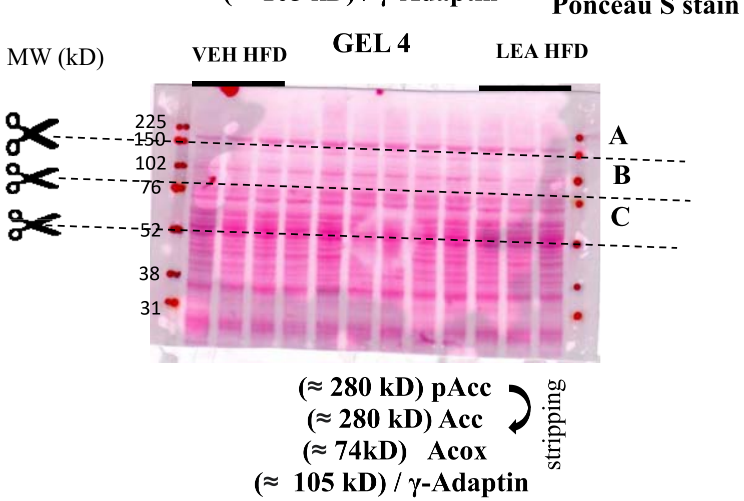

Whole membranes from Gel 1,2 .3and 4. Red

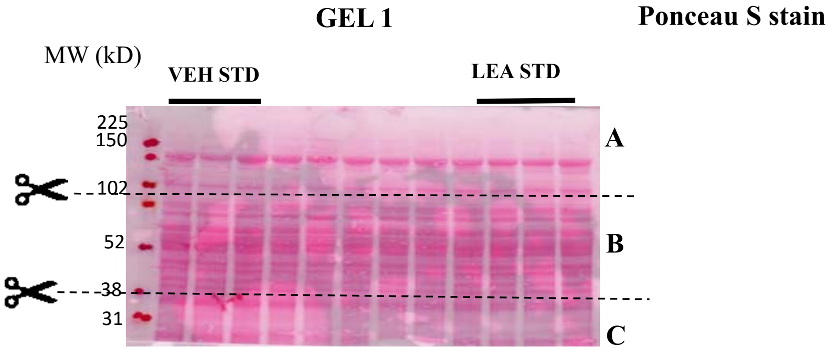

(≈ 52 kD) Pparα
(≈ 46kD) Nape pld
(≈ 105 kD) / γ-Adaptin

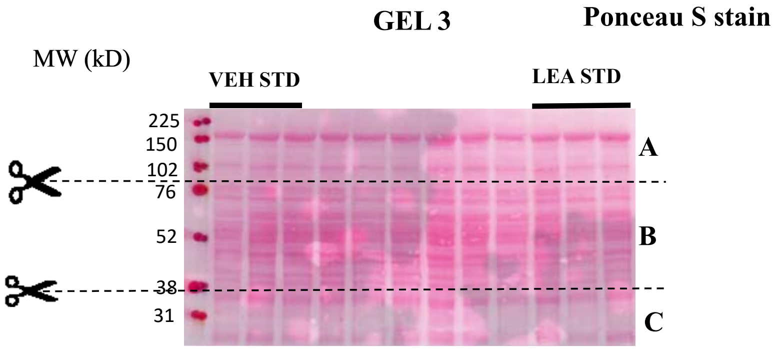

(≈ 52 kD) Pparα
(≈ 46kD) Nape pld
(≈ 105 kD) / γ-Adaptin

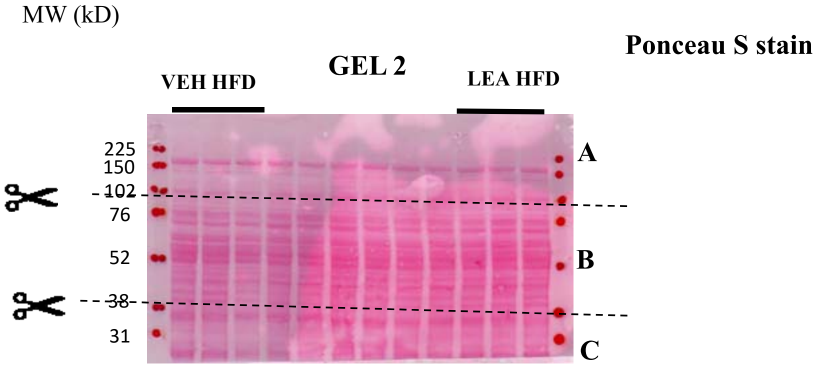

(≈ 52 kD) Pparα
(≈ 46kD) Nape pld
(≈ 105 kD) / γ-Adaptin

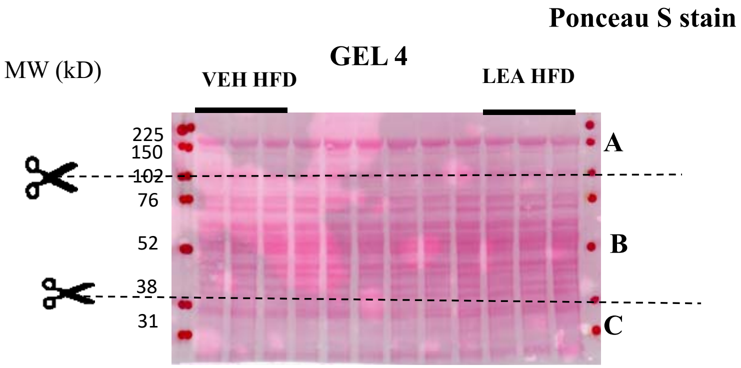

(≈ 52 kD) Pparα
(≈ 46kD) Nape pld
(≈ 105 kD) / γ-Adaptin
